# GPC3-Unc5 receptor complex structure and role in cell migration

**DOI:** 10.1016/j.cell.2022.09.025

**Published:** 2022-10-13

**Authors:** Onno Akkermans, Céline Delloye-Bourgeois, Claudia Peregrina, Maria Carrasquero-Ordaz, Maria Kokolaki, Miguel Berbeira-Santana, Matthieu Chavent, Florie Reynaud, Ritu Raj, Jon Agirre, Metin Aksu, Eleanor S. White, Edward Lowe, Dounia Ben Amar, Sofia Zaballa, Jiandong Huo, Irene Pakos, Patrick T.N. McCubbin, Davide Comoletti, Raymond J. Owens, Carol V. Robinson, Valérie Castellani, Daniel del Toro, Elena Seiradake

**Affiliations:** 1 Department of Biochemistry, University of Oxford, Oxford, UK; 2 MeLis, University of Lyon, Université Claude Bernard Lyon 1, CNRS UMR 5284, INSERM U1314, Institut NeuroMyoGène, 8 avenue Rockefeller 69008 Lyon, Lyon, France; 3 Department of Biological Sciences, Institute of Neurosciences, IDIBAPS, CIBERNED, University of Barcelona, Barcelona, Spain; 4 Kavli Institute for Nanoscience Discovery, University of Oxford, Oxford, UK; 5 Institut de Pharmacologie et Biologie Structurale, Université de Toulouse, Toulouse, France; 6 Department of Chemistry, University of Oxford, Oxford, UK; 7 York Structural Biology Laboratory, Department of Chemistry, University of York, York, UK; 8 Structural Biology, The Rosalind Franklin Institute, Harwell Science Campus, Didcot, UK; 9 Division of Structural Biology, University of Oxford, Oxford, UK; 10 Child Health Institute of New Jersey, New Brunswick, NJ 08901, USA; 11 School of Biological Sciences, Victoria University of Wellington, Wellington, New Zealand

**Keywords:** uncoordinated-5, Unc5, glypican-3, GPC3, crystallography, structural biology, cell guidance, cell migration, cortex development, neuroblastoma, stripe assay, nanobodies, surface plasmon resonance, UNC5A, UNC5B, UNC5C, UNC5D

## Abstract

Neural migration is a critical step during brain development that requires the interactions of cell-surface guidance receptors. Cancer cells often hijack these mechanisms to disseminate. Here, we reveal crystal structures of Uncoordinated-5 receptor D (Unc5D) in complex with morphogen receptor glypican-3 (GPC3), forming an octameric glycoprotein complex. In the complex, four Unc5D molecules pack into an antiparallel bundle, flanked by four GPC3 molecules. Central glycan-glycan interactions are formed by N-linked glycans emanating from GPC3 (N241 in human) and C-mannosylated tryptophans of the Unc5D thrombospondin-like domains. MD simulations, mass spectrometry and structure-based mutants validate the crystallographic data. Anti-GPC3 nanobodies enhance or weaken Unc5-GPC3 binding and, together with mutant proteins, show that Unc5/GPC3 guide migrating pyramidal neurons in the mouse cortex, and cancer cells in an embryonic xenograft neuroblastoma model. The results demonstrate a conserved structural mechanism of cell guidance, where finely balanced Unc5-GPC3 interactions regulate cell migration.

## Highlights

• Uncoordinated-5 receptor D (Unc5D) and glypican-3 (GPC3) form an octameric complex
• Anti-GPC3 nanobodies (Nano^glue^, Nano^break^) enhance or weaken Unc5-GPC3 binding
• Unc5/GPC3 signaling causes cell contact-repulsion *in vitro*
• *In vivo*, Unc5/GPC3 modulate the migration of cortical neurons and neuroblastoma cells

## Graphical abstract

**Figure:**
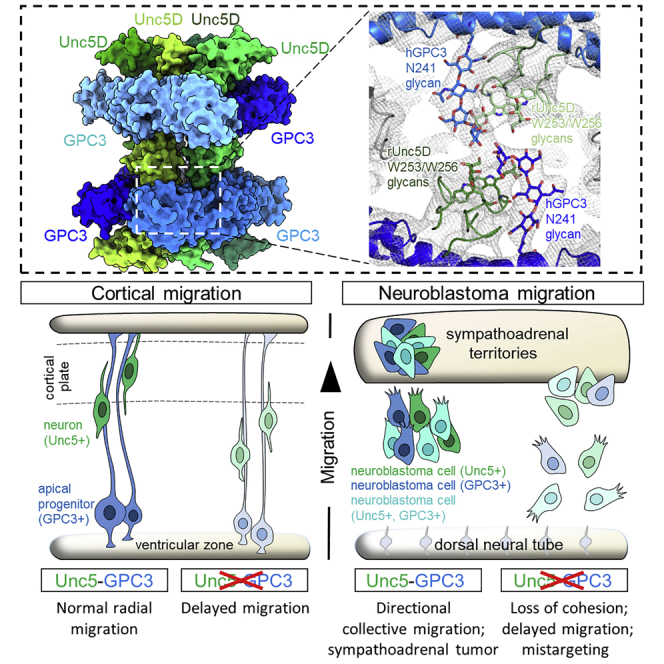

## Teaser

Detailed structural analysis and modulation of binding between uncoordinated-5 receptor D (Unc5D) and morphogen receptor glypican-3 (GPC3) reveal how finely balanced Unc5-GPC3 interactions regulate the migration of neuronal and cancer cells.

## Introduction

**Figure 1:**
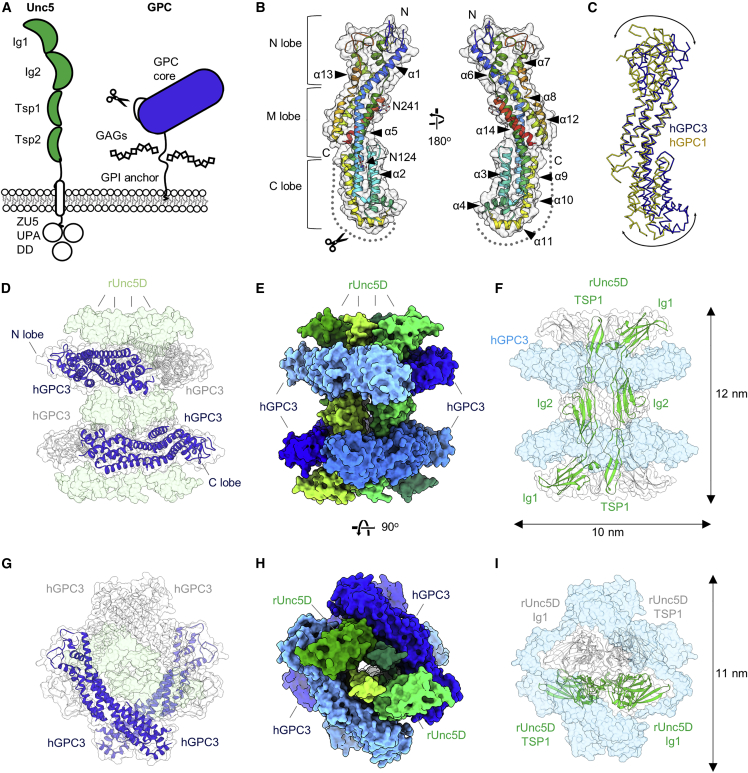
GPC3^core^ and Unc5^IgIgTSP^ crystal structures reveal an octameric complex (A) Domain architecture of Unc5 and GPC3. A furin-type cleavage site is indicated by a scissors symbol. (B) Crystal structure of hGPC3^core^, using the nomenclature presented in (Kim et al., 2011). The structure is colored according to the rainbow (blue, N terminus; red, C terminus). (C) Superposition of hGPC1, yellow (Awad et al., 2015), and hGPC3 core, blue. (D–I) Views of the hGPC3^core^-rUnc5D^IgIgTSP^ complex in two orientations, indicating hGPC3^core^ as ribbons in dark blue and gray (D and G), an overview of the complex in solid surface view with GPC3 in shades of blue and Unc5 in shades of green (E and H), and views in which the Unc5 chains are indicated in green and gray ribbons (F and I). Video S1 shows additional views. See also Figure S1, Document S1, Table S1, and Video S1.

Context-dependent signaling networks formed by different cell surface proteins direct brain development. Guidance receptors of the Uncoordinated-5 family (Unc5A–D) have emerged as key players in navigating cells and axons (Hong et al., 1999; Leung-Hagesteijn et al., 1992), where they trigger cell-cell repulsion in response to extracellular ligands such as fibronectin leucine-rich repeat transmembrane proteins (FLRT1-3) (Seiradake et al., 2014; Yamagishi et al., 2011) and netrins (Hong et al., 1999). Unc5D receptors guide neurons during radial migration, a key process that is required for the formation of functionally distinct cortical layers (Miyoshi and Fishell, 2012; Seiradake et al., 2014; Yamagishi et al., 2011). In this process, pyramidal neurons born from germinal layers are initially multipolar, as they move from the subventricular zone (SVZ) through the intermediate zone (IZ). In the upper IZ, these neurons transition to a bipolar morphology and attach to fibers of apical progenitor (AP) cells, enter the cortical plate (CP), and settle in their appropriate layer (Tabata and Nakajima, 2003). Unc5D is one of few molecular receptors known to regulate the switch from multipolar to bipolar migration. Altering Unc5D expression disrupts multipolar to bipolar transition, delays cortical migration, and affects layering of the mouse cortex (Miyoshi and Fishell, 2012). Netrin expression is low during radial migration, but FLRT2 is shed from cells in the CP, and prevents premature migration of Unc5D-expressing neurons (Yamagishi et al., 2011). Unc5 receptors consist of two extracellular immunoglobulin domains (Ig1–2), two extracellular thrombospondin-like domains (TSP1–2), a single transmembrane helix, and a C-terminal intracellular supramodule, which contains a ZO-1/Unc5 (ZU5), an Unc5/PIDD/Ankyrin (UPA), and a death domain (DD) (Figure 1A). We previously solved the ectodomain structure of human Unc5A isoform 1, which lacks TSP1 (Seiradake et al., 2014), and rat Unc5D Ig1-Ig2-TSP1 in complex with FLRT2 and Latrophilin 3 (Jackson et al., 2016). The structures revealed a linear arrangement of Unc5 Ig and TSP domains. FLRT binds at the N-terminal Ig1 domain. The crystal structure of the Unc5B ZU5-UPA-DD is also known and revealed a closed, autoinhibitory configuration (Wang et al., 2009).

In agreement with previous findings (Verschueren et al., 2020), we show that Unc5 receptors directly and functionally interact with the morphogen receptor glypican-3 (GPC3). All glypicans share a similar architecture: a structured N-terminal extracellular core domain, followed by a C-terminal linker region of ∼80 amino acids (Kim et al., 2011) (Figure 1A). Crystal structures of the core domain of human GPC1 (Awad et al., 2015; Svensson et al., 2012) and the fly ortholog Dally-like-protein (DLP) (Kim et al., 2011; McGough et al., 2020) revealed an α-helical architecture comprising an N-terminal lobe (N lobe) with 6 conserved disulfide bonds, a central M lobe, and a C-terminal lobe (C lobe) that contains a furin-like convertase cleavage site (RXXR) (de Cat et al., 2003). Cleavage of this site results in the formation of two fragments that remain covalently attached (de Cat et al., 2003). The C-terminal linker carries a glycosylphosphatidylinositol (GPI) anchor that tethers the protein to the cell surface, and is an attachment site for heparan sulfate (HS) glycans (David et al., 1990; Watanabe et al., 1995) (Figure 1A). HS glycans are sufficient for, or contribute to, the binding of many reported GPC3 interaction partners (Wang et al., 2020), such as Wnts, Frizzled, and Hedgehog (Capurro et al., 2005, 2008, 2014). Structures to show how these proteins interact with GPC3 have not been reported.

Mutations in GPC3 cause Simpson-Golabi-Behmel overgrowth syndrome (SGBS), a genetic disorder that presents with visceral and skeletal abnormalities and an increased risk of cancer (Cano-Gauci et al., 1999; Pilia et al., 1996; Tenorio et al., 2014; Veugelers et al., 2000). SGBS patients also display mild/moderate intellectual deficiencies and malformations of cortical development (Barkovich et al., 2012; Cottereau et al., 2013), which could suggest a role in brain development. GPC3 is a known regulator of apoptosis (Grisaru et al., 2001; Liu et al., 2012; Miao et al., 2013, 2014; Sun et al., 2011) with functions in hepatocellular carcinoma (Zheng et al., 2022). The expression of Unc5 receptors is affected in a variety of cancers (Mehlen and Guenebeaud, 2010), and is of prognostic value in neuroblastoma, where it drives tumor cell ability to migrate and/or survive (Delloye-Bourgeois et al., 2009; Wang et al., 2014). We recently developed the first relevant *in vivo* xenograft model, by grafting human neuroblastoma cells arising from the sympathoadrenal lineage of the neural crest into the equivalent site in chick embryos (Delloye-Bourgeois et al., 2017). Using this model, we demonstrated that neuroblastoma cells exploit semaphorin3c/neuropilin/plexin signaling, and use exogenous signals such as olfactomedin 1, for metastatic dissemination and to navigate a stereotypical migration pathway, which resembles that seen in patients (ben Amar et al., 2022; Delloye-Bourgeois et al., 2017). GPC3 is widely expressed in embryonal tumors (Ortiz et al., 2019) and was detected in a subset of aggressive neuroblastoma samples (Dong et al., 2020; Saikali and Sinnett, 2000).

Here, we present crystal structures that reveal a striking GPC3:Unc5D (4:4) octameric arrangement. Structured glycan-glycan interactions link C-mannosylated Unc5D tryptophans to an N-linked glycan on GPC3. Protein-protein interactions are formed along the concave face of GPC3 and all three N-terminal domains of Unc5D. We use mutagenesis and molecular dynamics simulations to characterize these interfaces, and present mutants that no longer interact. Anti-GPC3 nanobodies disrupt or enhance Unc5-binding. *In vitro*, we show that Unc5-GPC3 signaling elicits a repulsive cellular response. In the developing mouse cortex, we find that AP cells present GPC3 that acts as a ligand for neuronal Unc5D. We also show that Unc5/GPC3 signaling is essential for the collective migration of neural-crest derived neuroblastoma cells to their target sympathoadrenal derivatives.

## Results

### Structures of mouse and human GPC3

**Figure S1:**
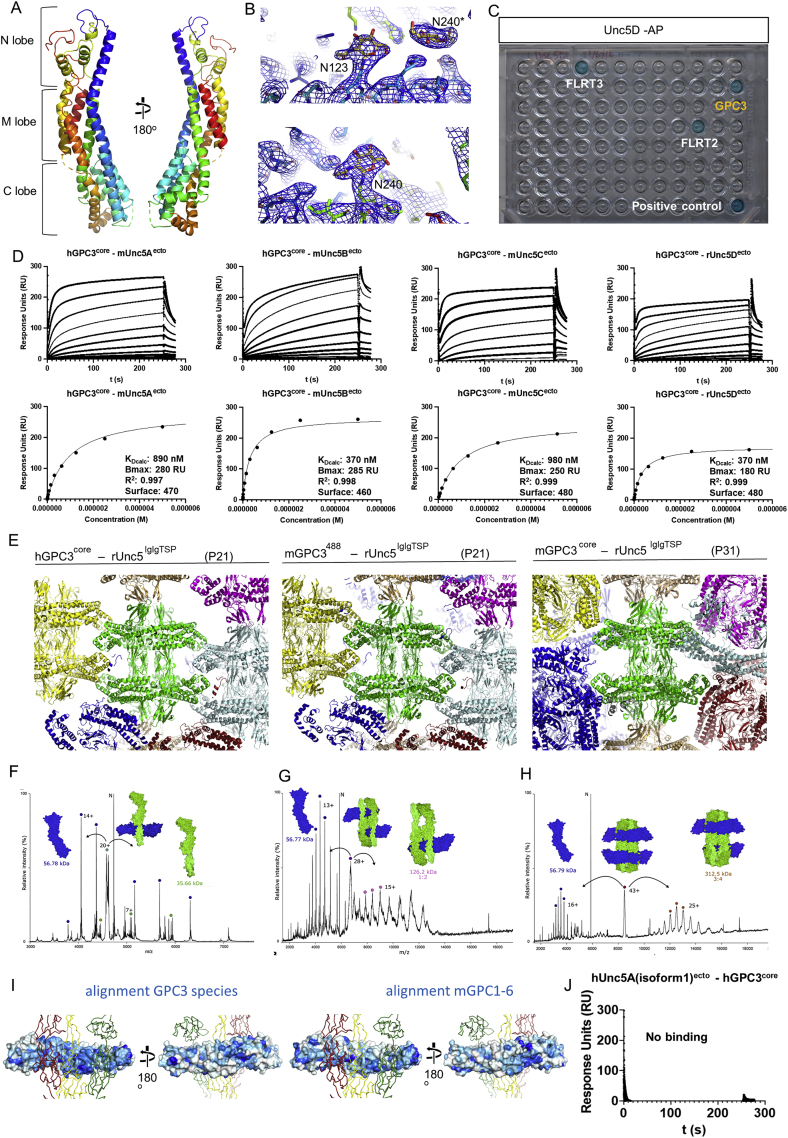
mGPC3^core^ structure and GPC3-Unc5D complex data, related to Figure 1 (A) Mouse GPC3^core^ structure colored according to the rainbow (blue: N-terminus, red: C-terminus). (B) Electron density map calculated from murine GPC3^core^ crystals is shown in blue, centered on N123 and N240 of a symmetry-related molecule^∗^ (left) and N240 (right). (C) ELISA plate contained Unc5D-AP (human, residues 33–379) immobilized in each well as bait and 95 other Fc-tagged proteins were added as prey, as described in (Ozgul et al., 2019). As expected, FLRT2/3 bind to Unc5D. GPC3 (human, residues 25–563) was a new positive interactor. Positive control was NRXN1-Fc/NLGN1-AP as described in (Ozgul et al., 2019). (D) SPR experiments show binding of Unc5 extracellular domains to hGPC3^core^. The apparent K_D_s (K_Dcalc_) were calculated using a 1:1 binding model and are indicative only. Bmax, R^2^ and amount of ligand immobilised on the flowcell surface are indicated. (E) Crystal packing environment for the three complex structures. Each octameric unit is shown in a different color, with a central unit in green. (F–H) Tandem MS (MS/MS) analysis of peaks presented in Figure 2A. Peaks reveal rUnc5^IgIgTSP^ and hGPC3^core(R355A/R358A)^ subcomplexes. The 93 kDa peak dissociated into masses corresponding to GPC3 (56.78 kDa excluding glycans) and Unc5D (35.66 kDa excluding glycans), the peaks corresponding to 185 and 370 kDa dissociated into GPC3 (56.78 kDa) and a mass of 126 kDa (consistent with a 2:1 Unc5D:GPC3 complex). In the 370 kDa peak we additionally detected a 312 kDa species (consistent with a 4:3 Unc5D:GPC3 complex). Charge state series (labeled with colored dots) are assigned to the complexes shown. (I) rUnc5D^IgIgTSP^ is shown in red, yellow and green ribbons, as found in the complex with mGPC3^core^. The surface of mGPC3^core^ is colored in shades of blue according to sequence conservation (blue = conserved, while = not conserved). Surface conservation was calculated using aligned sequences from human, mouse, opossum, chicken, frog, and fish GPC3 (top) or mouse GPC1-6 (bottom). Note that the Unc5-binding site is less conserved amongst mouse GPC1-6 sequences, compared to different GPC3 sequences. (J) SPR results show that hUnc5A isoform A, which lacks a TSP1 domain, is unable to bind hGPC3^core^.

We solved crystal structures of GPC3 residues 32–483 (hGPC3^core^) and murine GPC3 residues 31–482 (mGPC3^core^). Human and murine GPC3 sequences are 94% identical and the two structures are similar (Cα root-mean-square deviation, RMSD_Cα_ = 0.54 Å for 353 aligned atoms; Figures 1B and S1A). Compared to previously solved structures of fly DLP and human GPC1, GPC3 has a more curved shape (Figure 1C). Superposition of hGPC1 (Awad et al., 2015) and hGPC3^core^ results in an RMSD_Cα_ = 8.87 Å (for 358 aligned atoms). We modeled glycans on two predicted N-glycosylation sites (N124 and N241 in hGPC3, Figure 1B, N123 and N240 in mGPC3) into evident electron density (Figure S1B). Crystallographic details are summarized in Table S1.

### GPC3 is a high-affinity ligand for Unc5 receptors and forms an octameric hetero-complex

During an unbiased enzyme-linked immunosorbent assay (ELISA) (Ozgul et al., 2019; Ranaivoson et al., 2019), we confirmed the interaction of Unc5D with FLRT2 and FLRT3 and identified GPC3 as a ligand for Unc5D (Figure S1C). We confirmed the interaction using surface plasmon resonance (SPR) binding experiments with purified ectodomains (Figure S1D). To produce complex crystals, we mixed hGPC3^core^, mGPC3^core^ or murine GPC3 residues 31–488 (mGPC3^488^) with *Rattus norvegicus* Unc5D residues 32–307 (rUnc5D^IgIgTSP^). The complexes crystallized in two different space groups (Table S1) with different crystal packing. Strikingly, all three datasets revealed an octameric assembly (Figures 1D–1I, S1E, and Video S1). The center of the octamer is formed by four Unc5D molecules that are aligned in a “head-to-tail” antiparallel bundle. Two GPC3 molecules wrap around each end of the Unc5 tetramer. Each GPC3 chain interacts with three Unc5 chains, forming interfaces with the Ig1/Ig2 domains of two Unc5 molecules, and with a TSP1 domain of a third Unc5 molecule (Figures 1D–1I).

Video S1. Rotating view of the hGPC3core + rUnc5IgIgTSP complex crystal structure, related to Figure 1 hGPC3^core^ is shown in shades of blue, rUnc^5IgIgTSP^ is shown in shades of green.

**Figure 2:**
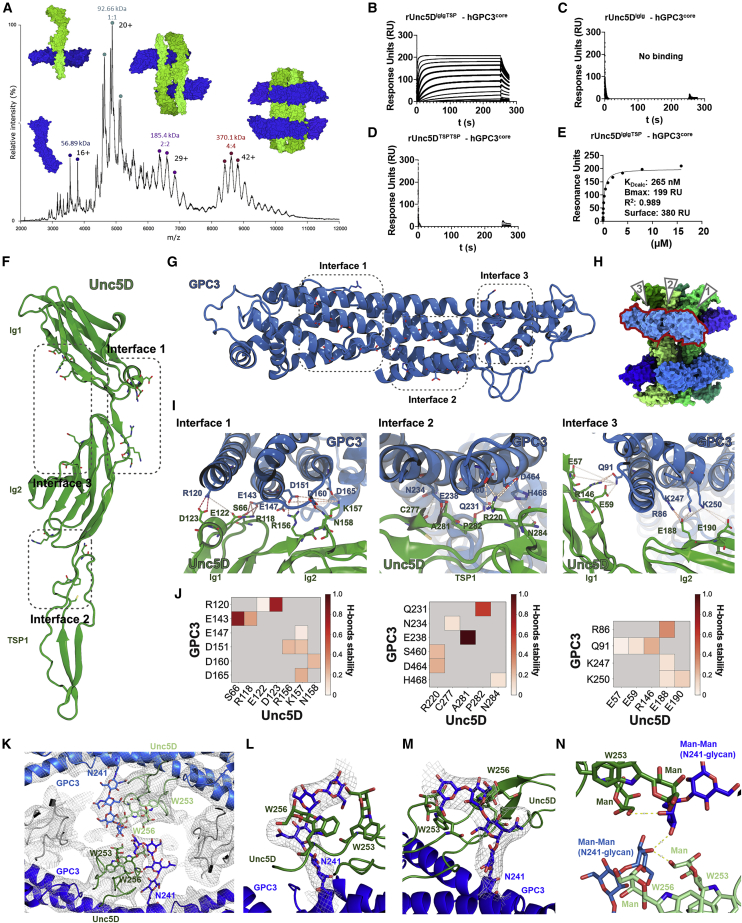
Characterization of the hGPC3-rUnc5 complex (A) Native MS spectrum of rUnc5^IgIgTSP^ and hGPC3^core^ (R355A/R358A). Charge state series (labeled with colored dots) are assigned to the complexes shown. Individual peaks were isolated for MS/MS analysis to identify subcomplexes (Figures S1F–S1H). (B–E) SPR data shows binding that rUnc5^IgIgTSP^, but not the shorter constructs rUnc5D^IgIg^ and rUnc5D^TSPTSP^, binds hGPC3core with nanomolar affinity. The apparent K_D_ (K_Dcalc_) was calculated using a 1:1 binding model and is indicative only. (F) Binding interfaces 1–3 on rUnc5D^IgIgTSP^. (G) Binding interfaces 1–3 on hGPC3^core^. (H) Binding interfaces 1–3 indicated on the octameric complex. The glypican molecule for which these are shown is outlined in red. (I) Zoomed views of interacting residues in interfaces 1–3 (hGPC3^core^-rUnc5D^IgIgTSP^ complex). Hydrogen bonds are shown as dotted yellow lines. (J) Summaries of the hydrogen bond analyses during restrained molecular dynamics (MD) simulation. Atoms that contribute to stable hydrogen bonds between the two proteins are shown, and colored blocks indicate the stability of the bond during simulation (averages for the four copies of the complex). Non-averaged results are shown in Figures S2A–S2C. (K) View of the glycan emanating from two copies of hGPC3 N241 toward the center of the complex. C-mannosylated tryptophans of nearby rUnc5D TSP1 domains are indicated (W253, W256). The calculated 2FoFc map of the hGPC3-rUnc5D complex data is shown as a gray mesh (sigma = 1). (L and M) As (K), but showing zoomed views of the N241-glycan for one of the hGPC3 copies within the complex. The map is carved around the N-linked glycan. (N) Distances below 3.5 Å between atoms within glycans from different chains are indicated as yellow dotted lines. See also Figure S2.

We performed native mass spectrometry to assess the stoichiometry of the complex outside a crystal lattice. Wild type (WT) GPC3 protein did not give clean signals using this method. We speculated that this may be due to a mixture of cleaved and uncleaved protein in our samples, which is only partially processed at the conserved furin-like convertase site in the C-lobe. To produce a more homogeneous sample for mass spectrometry, we introduced two-point mutations in the furin cleavage site (R355A, R358A). Mixed with rUnc5D^IgIgTSP^, we revealed masses corresponding to the octamer and its subfragments: 1:1 Unc5D-Gpc3 dimers (93 kDa), 2:2 tetramers (185 kDa) and the full 4:4 octamer (370 kDa) (Figure 2A). Each peak was subjected to tandem mass spectrometry (MS/MS) to validate the peak components (Figures S1F–S1H). These results support our structural conclusions.

### The GPC3-Unc5 super-complex requires multiple binding surfaces

**Figure S2:**
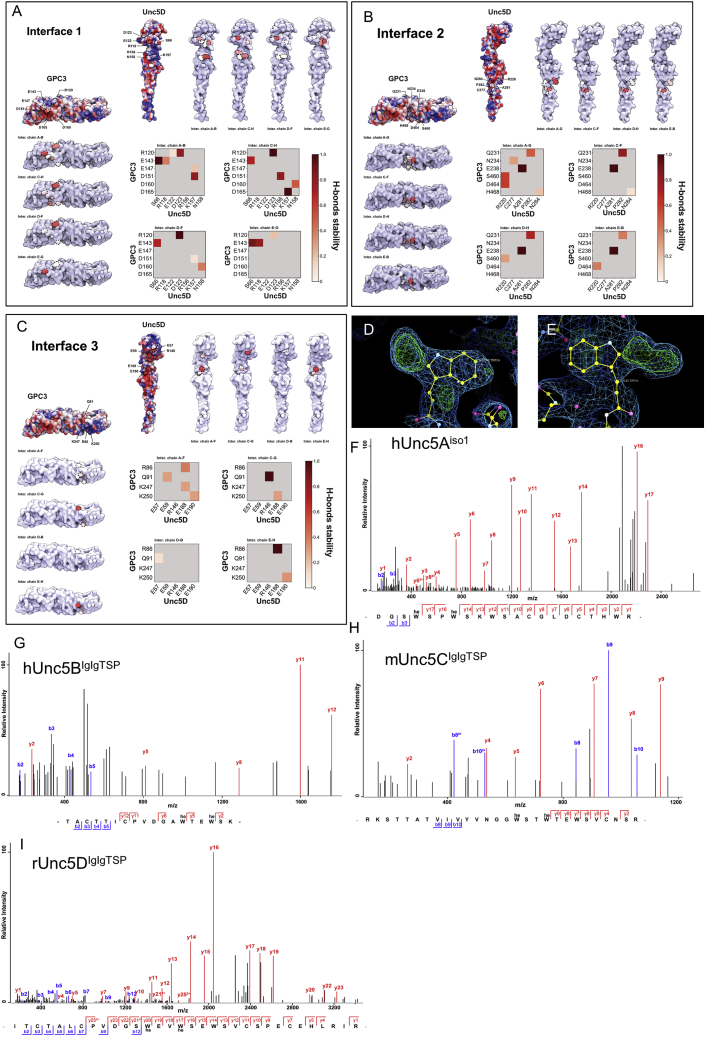
Hydrogen bond analysis during MD simulation and mass spectrometry analysis of Unc5 peptides, related to Figure 2 (A–C) Quantification of MD simulation results for each of the four pseudo-symmetrical copies in the complex, for each of the three rUnc5D-hGPC3 interfaces described in Figure 2. (D and E) Views of the electron density maps calculated for the X-ray crystal structure of Unc5Aiso1 (Seiradake et al., 2014): the 2Fo-Fc is shown in blue (1 sigma level). The Fo-Fc map is shown in red/green (+/− 3 sigma level). Extra density is observed on the first two of the TSP tryptophans of the consensus W1xxW2xxW3 motif. (F–I) LCMSMS of the tryptic Unc5 peptides confirming the C-mannosylation of tryptophan residues in the TSP1 domains of Unc5 proteins expressed in HEK cells.

The octameric arrangement of the complex involves three main interfaces, in which the highly sequence-conserved concave surface of GPC3^core^ contacts the three N-terminal domains of Unc5: Ig1, Ig2, and TSP1 (Figure S1I). SPR experiments show that both the Ig domains alone, or the TSP domains alone, are not sufficient for detectable binding to GPC3 (Figures 2B–2E). This arrangement contrasts with that of Unc5-FLRT complexes, where a single interface forms between the FLRT leucine-rich repeat (LRR) domain and Unc5 Ig1 (Jackson et al., 2016; Seiradake et al., 2014). In agreement with our conclusions, the human isoform 1 of Unc5A, which lacks TSP1 and therefore incudes only Ig1, Ig2, and TSP2 (hUnc5A^iso1^), is unable to bind GPC3 (Figure S1J). Unc5 sequence alignments show that conserved GPC3-binding residues are missing in this isoform (Document S1). To better characterize the protein-protein binding interfaces, we performed 500 ns of molecular dynamics simulations of the hGPC3^core^-rUnc5D^IgIgTSP^ complex. Averaged stable interactions at the main interfaces (Figures 2F–2H) are shown in Figures 2I and 2J. Equivalent data for each copy in the octamer is shown in Figures S2A–S2C.

Interface 1 is located at the C lobe of GPC3 and interfaces with the Ig1 and Ig2 domains of Unc5D, burying a surface of ∼690 Å^2^ (mGPC3 complex) or ∼640 Å^2^ (hGPC3 complex). Charge complementarity of the interface is provided by hGPC3 (R120, E143, E147, D151, D160, D165) and rUnc5D (R118, E122, D123, R156, K157) (Figures 2I, 2J, and S2A). The largest interface, 2, is formed between the Unc5D TSP1/Ig2 domains and the M lobe of GPC3, burying ∼1440 Å^2^ (mGPC3 complex) or ∼1330 Å^2^ (hGPC3 complex) of protein surface. Hydrophobic residues line both sides: rUnc5D I170, A281, P282, L283, F288, and hGPC3 L157, L235. Hydrophobic interactions are complemented by hydrogen bonding and charged interactions, such as hGPC3 E238, which interacts with the backbone of rUnc5D A281 (Figures 2I, 2J, and S2B). Interface 3 involves the N lobe of GPC3 and contains many charged and hydrogen-bonding interactions (Figures 2I, 2J, and S2C). The interacting surfaces are contributed by distinct patches on Unc5D, located on Ig1 and Ig2. The buried surface is ∼390 Å^2^ (mGPC3 complex) or ∼360 Å^2^ (hGPC3 complex). Within the octameric arrangement, the antiparallel Unc5D chains also form interactions between themselves, especially at the Ig2 domains: the buried surface amounts to ∼900 Å^2^ and ∼840 Å^2^ for the major antiparallel packing interactions within the Unc5 bundle.

### The Unc5-GPC3 complex is stabilized by an essential inter-chain glycan interaction

Recent work showed that the TSP domains of Unc5 receptors are C-mannosylated at tryptophan residues W_1_ and W_2_ of the consensus sequence W_1_xxW_2_xxW_3_ (Shcherbakova et al., 2019). We revisited our 2.4 Å-resolution published crystallographic data for hUnc5A^iso^ (Seiradake et al., 2014) and noticed evidence for C-mannosylation (Trp245 and Trp248), Figures S2D and S2E. We purified hUnc5A^iso1^, hUnc5B^IgIgTSP^ (residues 26–303), mUnc5C^IgIgTSP^ (residues 40–317), and rUnc5D^IgIgTSP^ from HEK293T cells. Mass spectrometry shows that the first two tryptophan residues (W_1_ and W_2_) were C-mannosylated in all Unc5 homologues tested (Figures S2F–S2I). In agreement with these results, we observed electron density for these glycans in the crystallographic maps.

**Figure 3:**
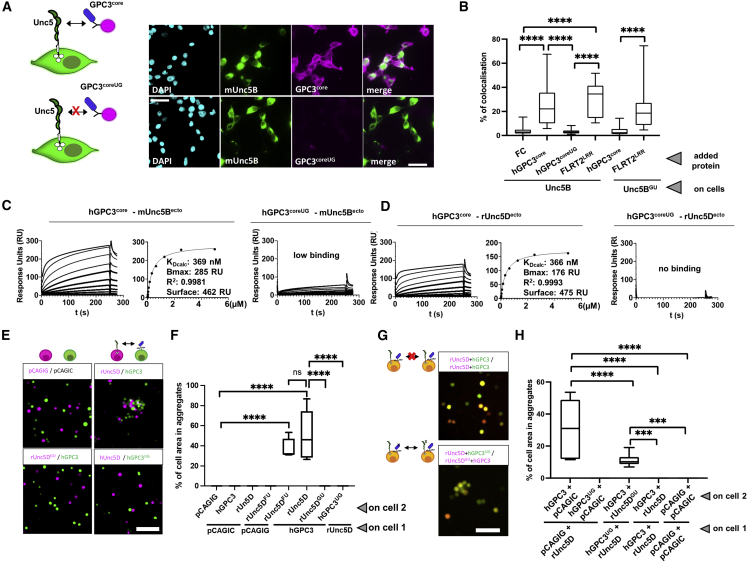
The non-binding mutants Unc5^GU^ and GPC3^UG^, Unc5-GPC3 interaction “*in trans*” is inhibited by “*in cis*” interactions (A) A cell-based assay shows binding between mUnc5B (expressed on cells) and purified GPC3^core^, but not GPC3^coreUG^. Representative images. (B) Quantification of cell-based assays. The Unc5-lingand FLRT2 (LRR domain, FLRT2^LRR^) is also used. (C) SPR binding data using Unc5B^ecto^. The apparent K_D_ (K_Dcalc_) was calculated using a 1:1 binding model and is indicative only. (D) As (C), using rUnc5D^ecto^. (E) A cell-cell aggregation assay shows that GPC3 and Unc5D mediate cell adhesion in *trans.* Representative images. (F) Quantification of cell-cell aggregation experiments. Empty vector controls: pCAGIC (red) and pCAGIG (green). (G) Cell-cell aggregation assay using co-expression of Unc5D and GPC3 *in cis.* Representative images. (H) Quantification of experiments using co-expression. ^∗∗∗^p < 0.001; ^∗∗∗∗^p < 0.0001, one-way ANOVA with Tukey’s post hoc tests. Scale bars represent 100 μm. See also Figure S3.

**Figure S3:**
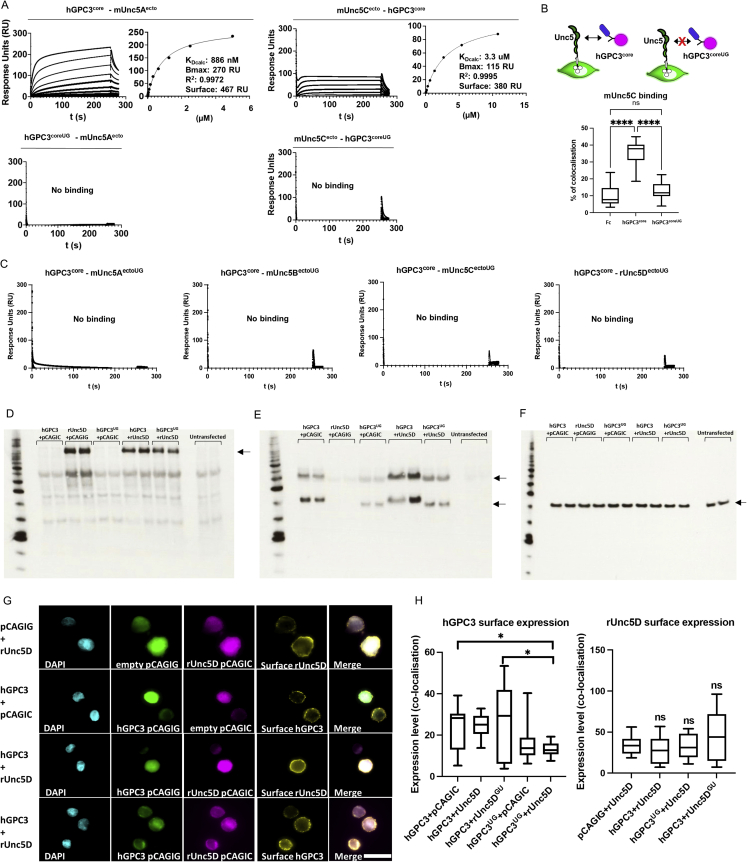
mUnc5A and mUnc5C binding results, protein co-expression analysis, related to Figure 3 (A) SPR results show binding of hGPC3^core^ protein to mouse Unc5A and C ectodomain. The apparent K_D_ (K_Dcalc_) for the wild type protein interaction was calculated using a 1:1 binding model and is indicative only. Bmax, R^2^ and the units of ligand immobilised on the flowcell surface are indicated. The N241Q mutant protein (hGPC3^coreUG^) does not show binding. (B) We used a cell-based assay to show that hGPC3^core^, but not the mutant, binds to mUnc5C expressed on cells. (C) SPR results show no binding of hGPC3^core^ protein to Unc5(A)–(D)^GU^ mutant proteins. Corresponding binding curves using wild type Unc5 proteins are shown in Figure S1D. (D) Western blot analysis using anti-HA to visualise HA-tagged Unc5 constructs expressed in cell aggregation assays. (E) Same samples as in panel D, but here visualising Flag-GPC3. (F) Same as panel D, but using anti-actin control. (G) Cell-surface staining using anti-HA and anti-Flag was performed to complement the total protein expression analysis shown in panels (D)–(F), and to include additional conditions. Representative images are shown. Scale bars = 30 μm. (H) Quantification of the experiments shown in panel F.

N-linked glycan chains are flexible and usually not defined in crystal structures unless they are held in place by specific interactions. The electron density maps calculated for the GPC3-Unc5D complexes revealed extra density extending from hGPC3 N241, one of the predicted N-linked glycosylation sites, toward the center of the complex. This glycan packs closely against C-mannosylated tryptophans 253 and 256 of rUnc5D TSP1 (Figures 2K–2N). We sought to remove this glycan *in vitro* to test its function. Attempts to remove it using endoglycosidases (EndoF1 or PNGase F) were not successful, as the glycan remained uncleaved. We therefore mutated the site (N241Q) to produce a hGPC3 protein that lacks a glycan at this position. The resulting mutant protein was readily expressed and secreted by HEK293 cells. A cell-based binding assay and SPR experiments showed that the N241Q mutant had lost affinity for Unc5A–D (Figures 3A–3D, S3A, and S3B). To be consistent with previously used nomenclature we will refer to this mutant as GPC3^UG^, with UG standing for “non-Unc5-binding GPC3”, throughout the rest of this manuscript. To produce non-GPC3 mutants of Unc5, we used an established approach where an artificial N-linked glycosylation site is engineered to disrupt protein-interaction (Jackson et al., 2015, 2016, 2018; Seiradake et al., 2013, 2014; del Toro et al., 2020) (Figure 3B). These Unc5 mutants contain a mutation in binding interface 2 (A277N + L279T in hUnc5B) and still bind the canonical ligand FLRT2 but not GPC3 (Figures 3B and 3S3C). In analogy to previous nomenclature, we call the resulting non-GPC3 binding mutants: Unc5^GU^.

### GPC3-Unc5D binding promotes cell-cell *‘in trans’* interaction

The geometry of the octameric complex begs the question whether these proteins interact on the surface of the same cells “*in cis*” or across cells “*in trans*”. We used an established cell aggregation assay using K562 cells (Berns et al., 2018; Pederick et al., 2021; del Toro et al., 2020) to assess “*trans*” interactions. The protein constructs used in this assay are “full length” and therefore anchored at the cell surface. We found that Unc5D-expressing cells bind and aggregate with GPC3-expressing cells *in vitro* (Figures 3E and 3F). The cells did not aggregate when we replaced the WT proteins with either rUnc5D^GU^ or hGPC3^UG^. Conversely, the non-FLRT binding rUnc5D (rUnc5D^FU^;Seiradake et al., 2014) causes aggregation with GPC3-expressing cells, confirming that the affected binding sites are distinct (Figures 3E and 3F), as supported also by the structural data. Next, we co-expressed Unc5D and GPC3 on the same population of cells to test whether *in cis* binding interferes with the *trans* interaction, as seen for other receptors (Carvalho et al., 2006). We found that cells co-expressing rUnc5D and hGPC3 did not aggregate (Figures 3G and 3H), indicating that *in cis*-interaction silences *trans* binding. In agreement with this finding, co-expression of WT rUnc5D + hGPC3^UG^ on one cell population and WT hGPC3 + rUnc5D^GU^ on the other population led to aggregation, showing that *in cis* interaction, rather than just co-expression, is required for silencing. *In cis* silencing can occur due to sequestering of binding surfaces on the cell surface, or due to inhibition of cell surface presentation of complexed proteins. We quantified the expression of the receptors using western blot analysis of whole cells and by cell surface immunostaining. The results demonstrated that co-expression does not prevent cell surface presentation of the receptors (Figures S3D–S3H). Taken together, the data suggest a mechanism by which the interaction can occur *in cis* or *in trans*. However, when the proteins bind *in cis*, then silencing of *trans* interaction occurs by occupying the available interaction sites.

### Characterization of functional anti-GPC3 nanobodies: Nano^glue^ and Nano^break^

**Figure 4:**
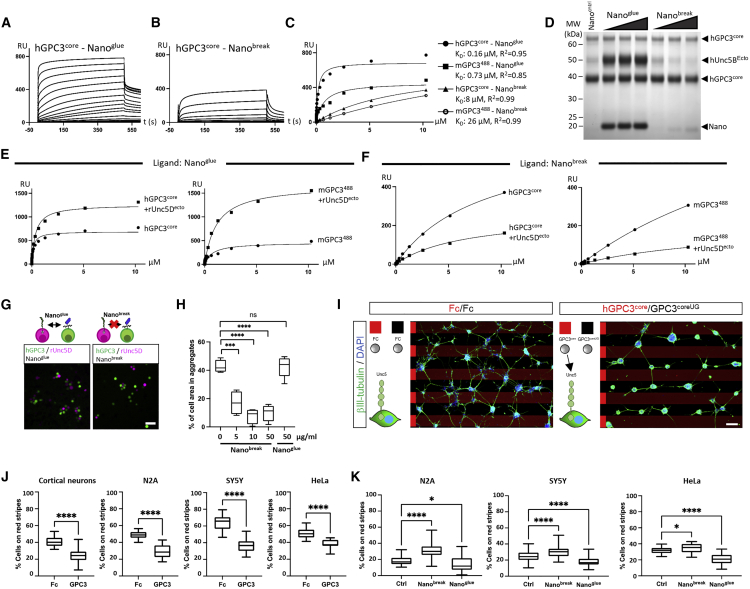
Nanobodies enhance or disrupt GPC3-Unc5 interaction (A) SPR binding data using hGPC3^core^ and Nano^glue^. (B) As in (A), with Nano^break^. (C) The equilibrium values from experiments shown in panels A and B, and equivalent values from using mGPC3^488^ (Figures S4E and S4F). K_D_ values were calculated assuming 1:1 binding. (D) Unc5B pull downs with immobilized hGPC3^core^ using Nano^glue^ and Nano^break^. (E and F) Equilibrium SPR data (raw data in Figures S4E and S4F) confirms that Nano^glue^ enhances, and Nano^break^ weakens, GPC3-Unc5D binding. (G) Cell-cell aggregation assay using soluble Nano^break^ and Nano^glue^. Representative images. (H) Quantification of cell-cell aggregation experiments. (I) E15.5 dissociated cortical neurons were grown on alternate stripes (red and black) containing Fc, GPC3^core^, or GPC3^coreUG^. (J) Quantification beta-III-tubulin+ (green) pixels on red stripes: Fc (=Fc/Fc control), GPC3 (=hGPC3^core^/hGPC3^coreUG^). We performed equivalent stripe assays also for HeLa, N2A and SY5Y cells, using DAPI for quantification. ^∗∗∗∗^p < 0.0001, Student T-tests. (K) Results from stripe assays in the presence of streptavidin (Ctrl) or streptavidin-nanobody complexes (Nano^glue^ or Nano^break^). We performed one-way ANOVA with Tukey’s post hoc tests (H) and (K). NS, not significant; ^∗^p < 0.05; ^∗∗∗^p < 0.001; ^∗∗∗∗^p < 0.0001. Scale bars represent 100 μm (G) and 90μm (I). See also Figure S4.

**Figure S4:**
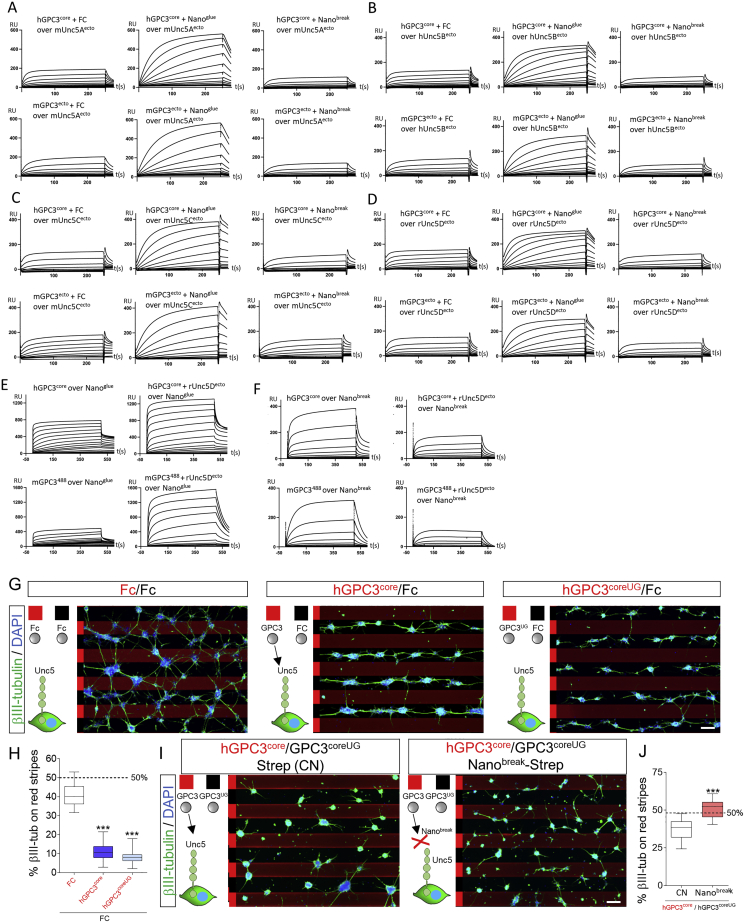
Nano^glue^ and Nano^break^ in SPR experiments and stripe assays, related to Figure 4 (A–D) Binding curves from SPR experiments. Unc5A-D receptor ectodomains were immobilised. Human GPC3^core^ or murine GPC3^ecto^ was injected using a 2-fold dilution series (top concentrations are 4.5 μM), in the presence of FC control protein, Nano^glue^ or Nano^break^. The concentration of nanobodies was kept constant at 9 μM (with hGPC3^core^), or 4.5 μM (with mGPC3^ecto^). The concentration of FC control protein was kept constant at equivalent mg/ml concentrations. (E and F) An analogous experiment was performed using immobilised nanobodies, and different concentrations of human GPC3^core^ or murine GPC3^488^ and Unc5D^ecto^. Taken together, the results demonstrate that Nano^break^ competes with Unc5 for GPC3-binding, whilst Nano^glue^ strengthens the interaction. Calculated K_D_s for nanobody-GPC3 interactions are shown in Figure 4C. Given the unusual stoichiometry of the Unc5-GPC3 complex, we have not calculated K_D_ values from experiments containing also Unc5. (G) Purified proteins were immobilised in a stripe pattern to assess their effect on the migration of cortical neurons. GPC3^core^ and GPC3^coreUG^ trigger strong cell repulsion, compared to neutral control protein (Fc). (H) Quantification of the experiments shown in panel F. One-way ANOVA with Tukey’s post hoc tests.^∗∗∗^p < 0.001. (I) We performed GPC3^core^/GPC3^coreUG^ stripe assays, but in the presence of streptavidin (CN) or streptavidin-nanobody complexes. Nano^break^ reduced the ability of neurons to distinguish between hGCP3^core^ and hGCP3^coreUG^. (J) Quantification of data shown in panel H. ^∗∗∗^p < 0.001, two-tailed Student’s T test. Scale bar represents 90 μm (G) and (I).

To generate additional tools for the functional characterization of the interaction, we characterized two llama-derived heavy-chain antibody-derived nanobodies, which bind to murine and human GPC3^core^ (Figures 4A–4C). Pull-down data suggests that one nanobody enhances complex formation between GPC3 and Unc5B (Nano^glue^), while another inhibits it (Nano^break^) (Figure 4D). We confirmed these results for Unc5A–D using SPR experiments (Figures 4E, 4F, and S4A–S4F). The pull down and SPR results also show that Nano^break^ has an overall weaker affinity compared to Nano^glue^. Consistent with the protein-binding studies, cell-cell aggregation assays showed that the addition of nanobody Nano^break^, and not Nano^glue^, inhibits Unc5D-GPC3 mediated cell-cell adhesion (Figures 4G and 4H). Of note, we did not observe enhanced aggregation with Nano^glue^ in this assay, which may be due to the strong aggregation phenotype observed, also in absence of the nanobody.

### GPC3-Unc5 interaction produces contact-repulsion *in vitro*

Unc5 receptors are known for their repulsive signaling in neuronal cell guidance (Round and Stein, 2007; Yamagishi et al., 2011). To assess if GPC3-Unc5 interaction is mediating contact-repulsion, we used stripe assays with cells known to express Unc5 receptors endogenously (Delloye-Bourgeois et al., 2009; Mehlen and Guenebeaud, 2010; Seiradake et al., 2014; Wang et al., 2014). Cells were plated on alternating stripes containing purified GPC3^core^ and the mutant GPC3^coreUG^. In these assays, cortical neurons preferentially migrated on the mutant protein stripes, demonstrating that GPC3^core^ elicits a repulsive effect via an Unc5-dependent mechanism (Figures 4I and 4J). Interestingly, when given the choice between GPC3^core^ (WT or mutant) and neutral Fc protein, the neurons were strongly repelled by both WT and the mutant protein. This suggests that unknown additional GPC3-receptors, who do not depend on the Unc5-GPC3 interaction, also cause repulsion from GPC3^core^ (Figures S4G and S4H). Further, we tested HeLa, N2A, and SY5Y neuroblastoma cell lines in stripe assays. As observed for the neurons, these cells preferred to grow on mutant, rather than WT, GPC3 protein (Figure 4J). We also tested the effects of nanobodies in these stripe assays. We tetramerized the nanobodies via a biotinylated linker and streptavidin to increase their affinity and potency. For N2A, SY5Y, and HeLa cells, the addition of nanobodies to the culture medium tended to enhance (Nano^glue^) or decrease (Nano^break^) cell repulsion from WT GPC3-containing stripes (Figure 4K), consistent with their functions to increase or decrease GPC3-Unc5 interactions, respectively. We attempted this assay with cortical neurons, however the addition of Nano^glue^ led to immobilization of neurons on the stripe surface and we were unable to quantify any migratory behavior (not shown). As for N2A, SY5Y, and HeLa cells, addition of Nano^break^ reduced the preference of neurons for GPC3^coreUG^ over GPC3^core^. We conclude that the GPC3-Unc5 interaction mediates contact-repulsion in these assays and may contribute to cellular navigation in cortical and neuroblastoma cells.

### GPC3 and Unc5D are expressed in the developing mouse brain cortex

**Figure 5:**
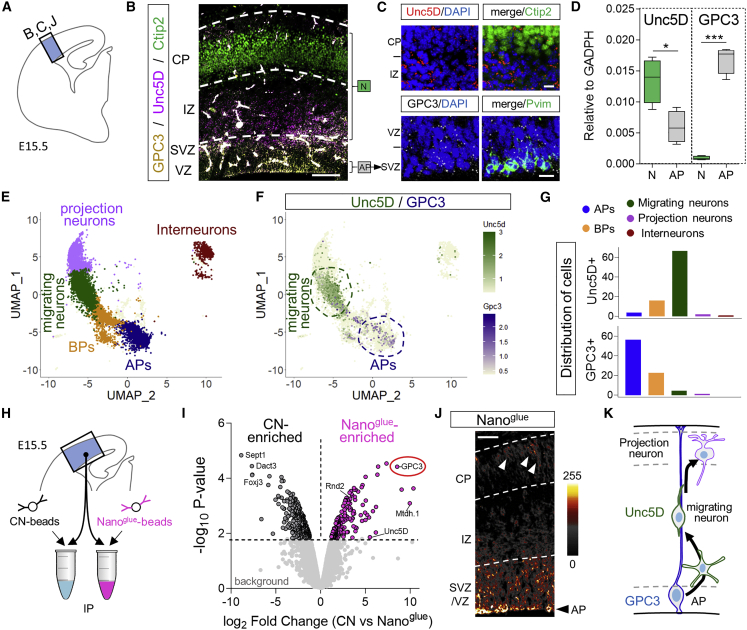
GPC3 is expressed by cortical apical progenitor cells (A) Cortical region shown in (B), (J), and magnified in (C). (B) Double *in situ* hybridization (ISH) for Unc5D (magenta) and GPC3 (yellow) shows their expression in the cortex at E15.5. ISH is combined with the neuronal marker Ctip2 (green). The layers enriched in neurons (N) and apical progenitors (AP) are indicated. (C) Upper panels show the ISH for Unc5D (red) combined with the neuronal marker Ctip2 (green). Lower panels show the ISH for GPC3 (white) and the apical progenitor marker Pvim (green). The location of the CP, IZ, SVZ, and VZ layers are indicated. Nuclear staining with DAPI is shown in blue. (D) Unc5D and GPC3 expression levels normalized to GADPH in neurons and APs, using RNA profiling data published in Florio et al. (2015) (GSE65000). Unc5D mRNA is high in neurons (N), while GPC3 mRNA is enriched in AP cells. ^∗^p < 0.05, ^∗∗∗^p < 0.001, two-tailed Student’s t test. (E) UMAP visualization of single-cell data from E15.5 mouse cortex published in di Bella et al., (2021) (GSE153164). Basal progenitor (BP). (F) Combined plot of Unc5D (green) and GPC3 (magenta) mRNA expression per cell. Most of Unc5D-expressing cells belong to the migrating neuron cluster (dashed green line), GPC3-expressing cells are enriched in the AP cluster (blue dashed line). (G) Quantification of the distribution of Unc5D- and GPC3-positive cells. (H) Cortical region used for pull down with Nano^glue^. (I) Volcano plot showing enriched proteins in control (black) and Nano^glue^ (pink) pull downs revealed by mass spectrometry. Non-significant proteins are represented in light gray. (J) Immunostaining for GPC3 using Nano^glue^ coupled to fluorescent streptavidin on coronal section of E15.5 mouse cortex. The image is colored based on the intensity of the staining. Arrowheads indicate staining that resembles the pattern of AP fibers. (K) Summary model showing Unc5 and GPC3 expression patterns. Scale bars represent 200 μm (B), (J, top left) and 20 μm (C). See also Figure S5.

**Figure S5:**
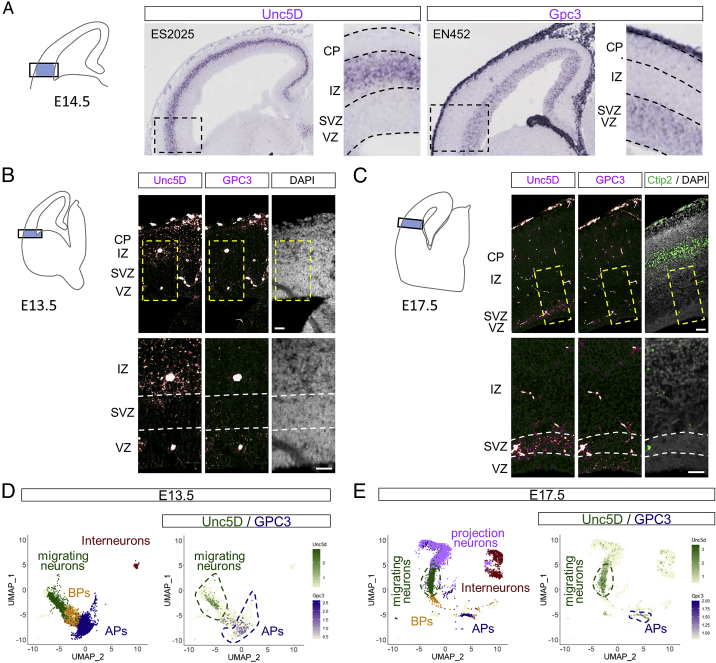
Unc5D and GPC3 are expressed during cortical development, related to Figure 5 (A) ISH for Unc5D and GPC3 in an E14.5 brain sagittal section. Unc5D is expressed in the IZ and GPC3 in the VZ/SVZ as indicated with higher magnification on the left. Images are from https://gp3.mpg.de and the image series ID is shown on the top left of each image. (B and C) ISH for Unc5D and GPC3, colored in magenta, shows expression in the cortex of coronal sections of E13.5 (B) and E17.5 (C) mouse embryos. Each panel shows a diagram on the left, which is indicating the cortical region shown. The area in the dashed rectangle is magnified on the bottom. (D and E) UMAP visualization of single-cell RNA sequencing data from E13.5 (D) and E17.5 (E) mouse cortex published in di Bella et al., (2021). Five major cell clusters, colored by cell-type assignment based on published metadata (GSE153164), are shown on the left. A combined plot of Unc5D (green) and GPC3 (magenta) mRNA expression per cell is shown on the right. Most of Unc5D-expressing cells belong to the migrating neuron cluster (dashed green line), while GPC3-expressing cells are highly enriched in the apical progenitor (AP) cluster (blue dashed line). Scale bars represent 100 μm (B) and (C).

Glypicans show specific patterns of expression during CNS development, with five out of six known glypicans (GPC1–4 and GPC6) expressed at earlier stages of brain development (Ford-Perriss et al., 2003) and in neural stem cells (Oikari et al., 2016). *In situ* hybridization (ISH) for GPC3 showed restricted expression to the germinal layers, predominantly at the ventricular zone (VZ) where AP cells are located from embryonic days (E)13.5 to E17.5 (Figures 5A, 5B, and S5A–S5C). Unc5D showed strong expression in areas enriched in young/migrating neurons (SVZ/IZ) as reported previously (Miyoshi and Fishell, 2012; Takemoto et al., 2011). These results were confirmed by co-staining with the neuronal marker Ctip2 and the AP marker Pvim (Figure 5C). Analysis using data from two single-cell RNA-seq databases showed that Unc5D is enriched in migrating neurons, whereas GPC3 is expressed predominantly in AP cells, from E13.5 to E17.5 (Florio et al., 2015; Figure 5D) (di Bella et al., 2021; Figures 5E,5F, S5D, and S5E). Distribution analysis using categorized clusters showed that 66% of Unc5D-positive cells are migrating neurons, whereas 57% of GPC3+ cells are APs at E15.5 (Figure 5G). Pull-down experiments using Nano^glue^ in E15.5 mouse cortex lysate led to enrichment of GPC3 protein. Moreover, Unc5D co-immunoprecipitated with GPC3, suggesting that the two proteins interact, at least in the context of this experiment (Figures 5H and 5I). Consistent with the expression data, we found that GPC3 protein is present in the germinal zone, mainly where the AP cell bodies are located within the VZ, and to a lower extent in the IZ and CP, where the pattern resembles that of AP fibers and their endfeet (Figure 5J). Based on these results, we developed a working model in which migrating neurons expressing Unc5D interact with GPC3 present in AP cells (Figure 5K).

### GPC3-Unc5 interaction is required for radial neuronal migration *in vivo*

**Figure 6:**
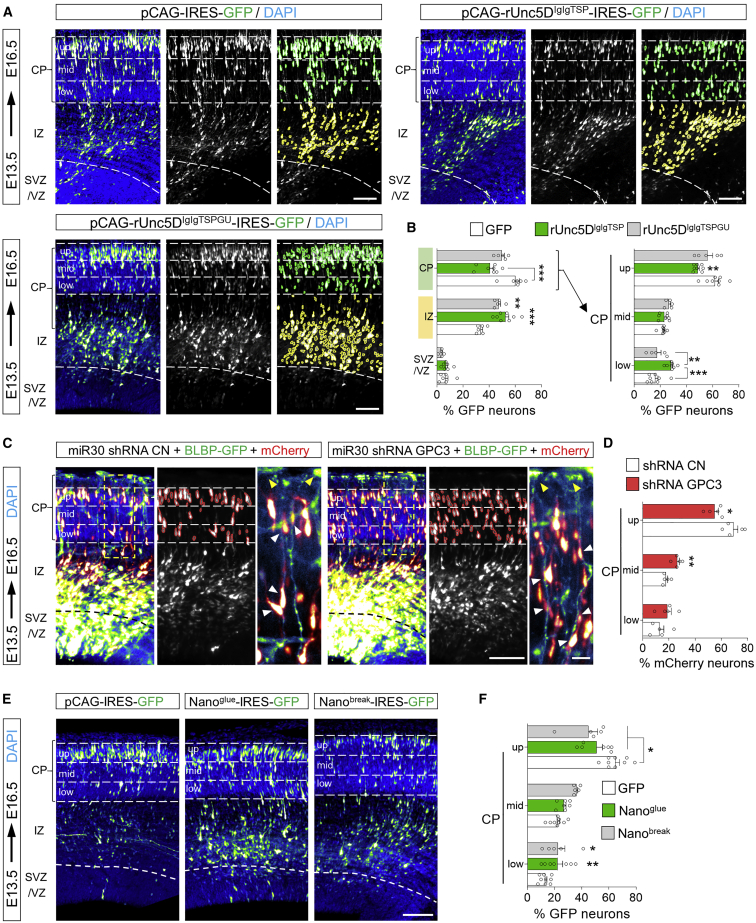
GPC3 promotes radial migration of Unc5-expressing cells (A) Coronal sections of E16.5 cortex after IUE using empty vector (pCAGIG, control), rUnc5^DIgIgTSP^, or rUnc5^DIgIgTSPGU^. The cortical plate (CP) is defined based on DAPI staining. GFP-positive cells in the IZ (yellow) and CP (green) are automatically identified and the percentage in each layer quantified. The CP is further subdivided into 3 bins (up, mid, low). (B) Quantification of data shown in (A). CP and IZ is highlighted in green and yellow, respectively. n = 8 GFP, n = 8 rUnc5^Decto^, and n = 5 rUnc5^DIgIgTSP^ electroporated brains. ^∗∗^p < 0.01, ^∗∗∗^p < 0.001, one-way ANOVA test with Tukey’s post hoc analysis. (C) Coronal sections of a E16.5 cortex electroporated with pCAGGS-mCherry, pCAG-BLBP-GFP and a pCAG-miR30 vector coding for shRNA control (CN) or shRNA targeting murine GPC3. The number of mCherry-positive neurons in contact with a GFP-positive radial fiber in each bin was quantified (white arrowheads, inset on the right). Endfeet of radial fibers are indicated with yellow arrowheads. (D) Quantification of the data shown in (C). n = 5 CN, n = 5 shRNA GPC3, electroporated brains. ^∗^p < 0.05, ^∗∗^p < 0.01, two-tailed Student’s t test. (E) Coronal sections of E16.5 cortex after IUE using empty vector (pCAGIG, control), Nano^glue^, or Nano^break^ at E13.5. GFP-positive were quantified for each bin. (F) Quantification of data shown in (E). n = 9 GFP, n = 7 Nano^glue^, and n = 5 Nano^break^ electroporated brains. ^∗^p < 0.05, ^∗∗^p < 0.01, one-way ANOVA test with Tukey’s post hoc analysis. Scale bars represent 100 μm (A),(C), and (E) and 20 μm (inset in C). See also Figure S6.

**Figure S6:**
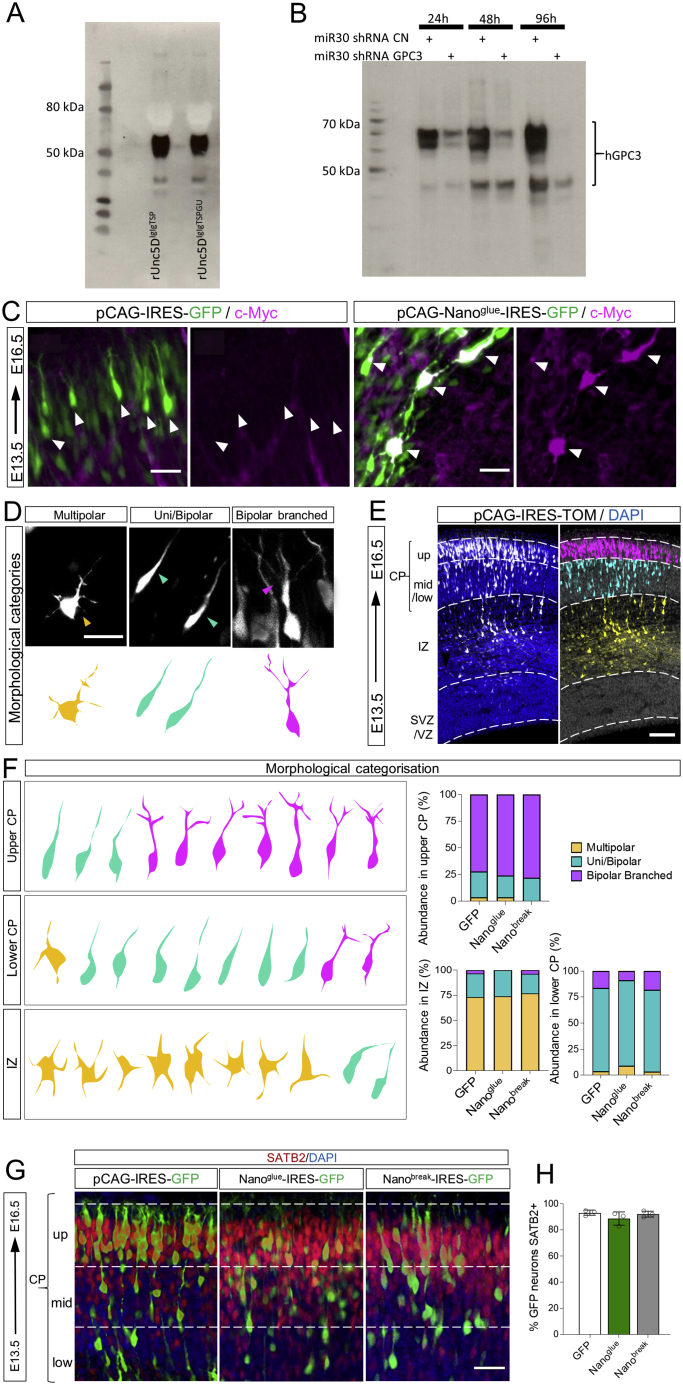
Validation of secreted Unc5D constructs and GPC3 shRNA *in vitro*, nanobody expression *in vivo*, related to Figure 6 (A) Anti-HA western blot showing the secretion levels of HA-tagged rUnc5^IgIgTSP^ constructs that we used in IUE experiments. Supernatants of transfected HEK293 cells were analyzed. We find that both constructs are secreted effectively. (B) Anti-FLAG blot showing hGPC3 expression in HEK cells, at different time points after transfection (24, 48, 96 h). HEK cells were co-transfected with vector expressing control (CN) or GPC3 shRNA. Significant reduction in GPC3 expression was observed after 24, 48 and 96h for cells co-transfected cells with GPC3 shRNA. Similar results were obtained for mGPC3^ecto^ (not shown), as expected, given that the target sequence is conserved in murine and human GPC3. (C) IUE of pCAG-IRES-GFP (pCAGIG, control) and pCAGIG encoding Nano^glue^-IRES-GFP was performed at E13.5 and analyzed at E16.5. Myc-tagged Nano^glue^ protein expression in neurons was confirmed by immunostaining with anti-Myc (magenta). Nano^glue^ expression coincides with the positions of cells expressing the reporter GFP (green). White arrows indicate neurons expressing GFP (control and Nano^glue^ plasmid). Scale bar represents 25mm. (D) We categorized neurons overexpressing nanobodies or GFP into multipolar, uni/bipolar, or bipolar branched phenotypes (example images). (E) Electroporated neurons in the upper CP (magenta), mid-lower CP (cyan) and IZ (yellow) are colored according to the highest abundance of each morphological category in each bin. Nuclear staining with DAPI is shown in blue. (F) Abundance of each category of neurons in the upper/mid-lower CP and in the IZ of electroporated brains. (G) Staining of electroporated slices (GFP, Nano^glue^ or Nano^break^) with the laminar marker Satb2 (red). Nuclear staining with DAPI is shown in blue. (H) Quantification of G. Scale bar represents 25mm (C), 20 μm (D), 100mm € and (G).

To study the effects of Unc5-GPC3 binding on cortical neuron migration, we used *in-utero* electroporation (IUE) at E13.5. We previously showed that the overexpression of signaling-deficient, but otherwise active, receptor fragments is an effective way of interfering with endogenous interactions (del Toro et al., 2020). Here, we over-expressed rUnc5D^IgIgTSP^ in migrating neurons (Figure 6A). Consistent with the role of Unc5D in radial migration, expression of the secreted ectodomain produced a strong delay in neuronal migration (Figures 6A and 6B). The accumulation observed in the IZ resembles the phenotype seen when full length Unc5D is over-expressed in migrating neurons (Seiradake et al., 2014; Yamagishi et al., 2011). This effect was partially rescued when using the mutant Unc5D^IgIgTSPGU^ (Figures 6A, 6B, and S6A), confirming that the migration delay is at least partially due to interaction with GPC3. In an alternative approach to reduce interactions, we knocked down endogenous GPC3 in E13.5 cortices using the small hairpin RNA (shRNA) target sequence in the pCAG-miR30 vector system (Matsuda and Cepko, 2007) (Figure S6B). We used the pCAG-BLBP vector (Shariati et al., 2013) to visualize the targeted AP cells and measured the distribution of WT neurons labeled with a mCherry reporter (Gurtan et al., 2012) (Figure 6C). Analysis at E16.5 showed reduced migration of neurons along the fibers deficient for endogenous GPC3 protein (Figures 6C and 6D). We also over-expressed secreted Nano^break^ and Nano^glue^ using the same IUE approach (Figure S6C). Both Nano^break^ and Nano^glue^ over-expression caused significant delays in migration to the upper CP (Figures 6E and 6F). When categorizing the neurons based on their morphologies (Figures S6D–S6E), we did not observe differences between Nano^glue^ and Nano^break^-overexpressing neurons and GFP controls (Figure S6F). Moreover, these neurons contain the differentiation marker Satb2 (Figures S6G and S6H), suggesting that the nanobodies affect the migration, but not the morphology/differentiation, of the cells. Taken together, these results show that Unc5-GPC3 interactions regulate cortical migration *in vivo*.

### GPC3-Unc5 interaction is required for neuroblastoma cell migration *in vivo*

**Figure 7:**
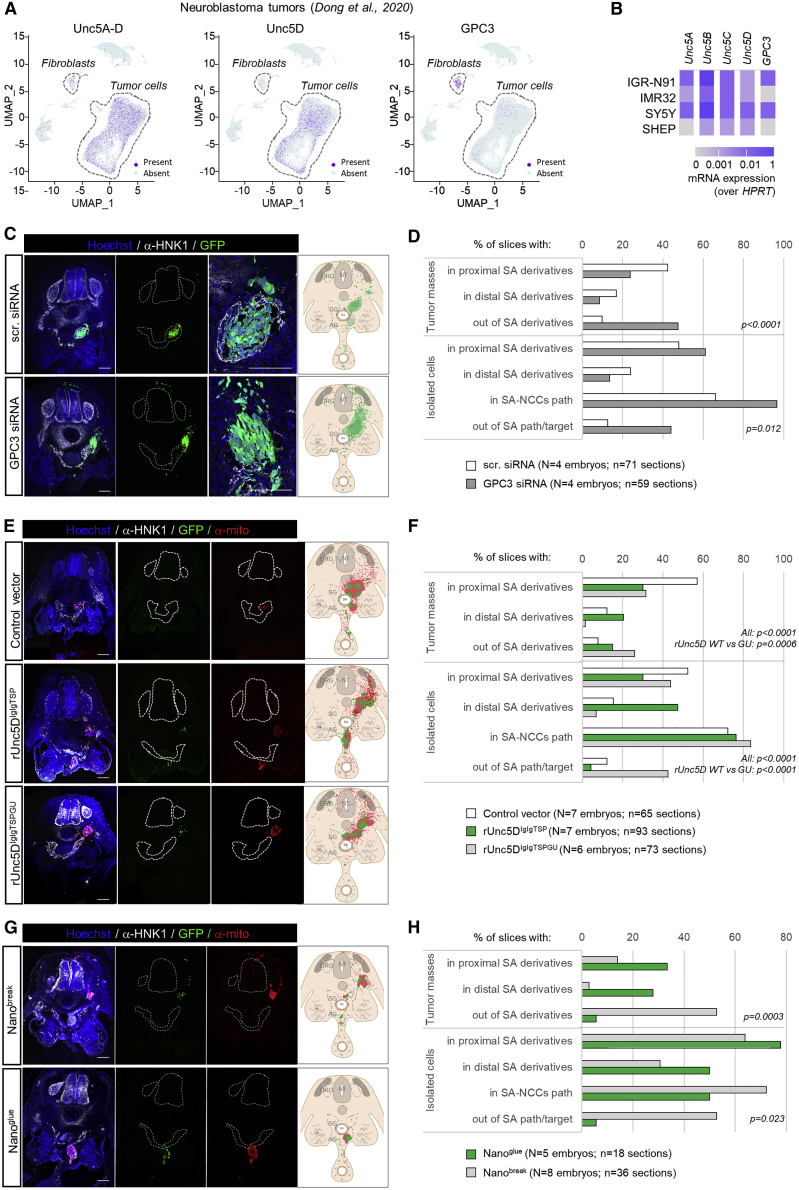
GPC3-Unc5 signaling determines neuroblastoma cell migration properties (A) UMAP visualization of single-cell data from neuroblastoma tumors (Dong et al., 2020) and quantification for selected transcripts. (B) Heatmap of Unc5A-D and GPC3 mRNA expression in 4 human neuroblastoma cell lines, measured by qRT-PCR. (C) SY5Y:GFP cells were transfected (scramble, scr., or GPC3 siRNA) and engrafted within the migratory trunk neural crest of E2 chicken embryos and slices analyzed 2 days later. Neural crest-derived structures were labeled with an anti-HNK1 antibody, nuclei with Hoechst. (D) Quantification of SY5Y cell and tumor positions two days after grafting. (E and F) As (C) and (D), but SY5Y cells were transfected with vectors encoding rUnc5D^IgIgTSP^, Unc5D^IgIgTSPGU^ or pCAGIG (control) vectors prior to the graft. Samples were labeled with anti-human mitochondrial antibody to reveal all SY5Y cells (transfected: green + red; non-transfected: red). (G and H) SY5Y cells were transfected with vectors encoding Nano^glue^ or Nano^break^ prior to the graft. Scale bars: 200 μm. For (D), (F), and (H), we used χ^2^ tests to compare scr. (control) versus GPC3 siRNA conditions (D), rUnc5d^ecto^ versus rUnc5d^ectoGU^ (F), and Nano^glue^ versus Nano^break^ conditions (H). NT: Neural Tube; S: Somite; DRG: Dorsal Root Ganglia; SG: Sympathetic Ganglia; AG: Adrenal Gland; DA: Dorsal Aorta; Me: Mesonephros. See also Figure S7.

**Figure S7:**
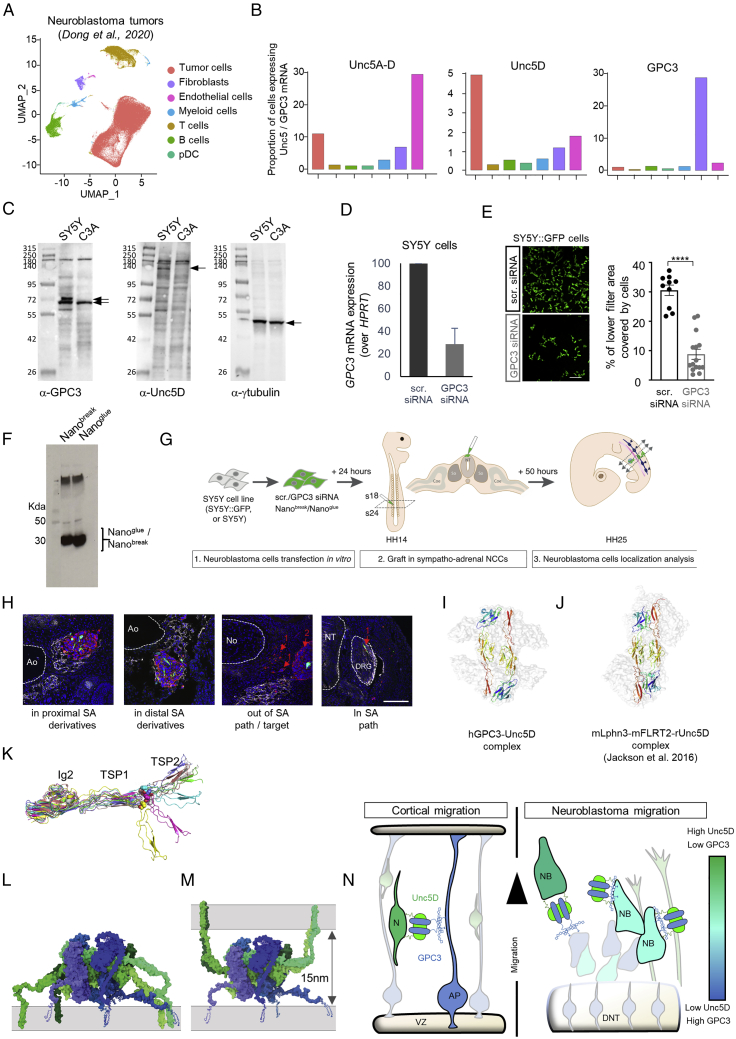
Interfering with GPC3-Unc5 interaction impacts on neuroblastoma cell migration properties; structural discussion of Unc5 complexes, related to Figure 7 (A) UMAP visualization of single-cell data from neuroblastoma tumors (Dong et al., 2020). (B) Quantification of Unc5A-D, Unc5D alone, and GPC3 transcripts for each cell type. (C) Western Blot analysis of GPC3 and Unc5D proteins in SY5Y and C3A cells. (D) Q-RT-PCR analysis of GPC3 mRNA expression in SY5Y cells, 24 h after transfection, using GPC3 siRNA or scr.siRNA as a control. (E) Representative images (left) and quantification (right) of transwell assays measuring the migratory properties of SY5Y:GFP cells transfected with either scr or GPC3 siRNA. ^∗∗∗∗^:p < 0.0001. Student T test with Welsch correction. (F) Anti-Myc western blot showing the secretion levels of Myc-tagged nanobody constructs used in Figure 7G. Supernatants of transfected SY5Y cells were analyzed. We find that both constructs are secreted effectively. (G) Scheme of the in ovo graft experimental paradigm describing the experiments presented in Figures 7C–7H. NB: neuroblastoma. (H) Illustrations of the phenotypic classification quantified in Figures 7C–7H. Neural crest-derived structures were labeled with an anti-HNK1 antibody. Human NB cells were detected with an anti-mito antibody (in red) and transfected NB cells with GFP (in green). Nuclei were stained with Hoechst. “1” points at isolated cells; “2” points at tumor masses. NT: Neural Tube; Ao: Dorsal aorta; DRG: Dorsal Root Ganglia; No: Notochord. Scale bar: 200 μm. (I) Two of the four rUnc5DIgIgTSP chains in the complex with hGPC3core are shown as ribbons, colored according to the rainbow (N-terminus = blue, C-terminus = red). The rest of the complex is shown as transparent surface (gray). (J) rUnc5DIgIgTSP in complex with FLRT2 and Latrophilin3 (Jackson et al., 2016). The two Unc5D chains are highlighted as rainbow ribbons. (K) Superpositions of Alpha-fold models of the rUnc5D Ig2-TSP1-TSP2 region, after MD simulation, suggests flexibility in the TSP1-TSP2 linker. (L) The ‘in cis’ model of hGPC3-rUnc5D was created using Alpha-fold, MD simulation and MODELLER. GPC3: shades of blue, Unc5D: shades of green. We have not included intracellular domains. (M) As panel L, but showing a potential ‘in trans’ configuration where GPC3 and Unc5D are expressed on adjacent cells. (N) Schematic summarizing the Unc5/GPC3 expression levels and putative interactions in the cortical and neuroblastoma models presented in this manuscript. Expression of Unc5D and GPC3 is color-coded from green (high Unc5D/ low GPC3) to blue (low Unc5D, high GPC3). Cells colored in cyan indicate co-expression of both receptors. N: neuron, AP: apical progenitor, VZ: ventricular zone, NB: neuroblastoma cell, DNT: dorsal neural tube

The phenotypes observed in cortical migration, together with the widely documented roles of Unc5 and GPC3 in cancer, led us to investigate whether Unc5/GPC3 interaction plays a role in neuroblastoma cell migration. We analyzed the expression of GPC3 and Unc5 in published single cell RNA-seq data from 16 different neuroblastoma patient samples (Dong et al., 2020). Unsupervised clustering of patients’ cell data led to a segregation of tumor cells from those of the stroma (Figures 7A and 7S7A). The fraction of tumor cells expressing at least one Unc5 receptor was higher in the tumor cell cluster as compared to other cell types (Figures S7A and S7B). Endothelial cells also highly express Unc5 receptors, especially Unc5B, as shown by others (Larrivée et al., 2007; Lu et al., 2004). Conversely, although GPC3 was detected in a fraction of tumor cells, its expression was more frequent (29%) in fibroblastic cells of the tumor microenvironment (Figure 7A). As found for the patient tumor cells, different neuroblastoma cell lines also expressed Unc5 receptors and some GPC3 (Figure 7B). We selected the SY5Y cell line to further study potential roles of GPC3-Unc5 interactions in neuroblastoma cell migration. GPC3 and Unc5 receptor proteins have been detected in these cells at the protein level (Delloye-Bourgeois et al., 2009; Heidler et al., 2018) and we verified their expression by western blot (Figure S7C). Human-specific GPC3 small interfering RNA (siRNA) reduced the expression of GPC3 by 71% (±11%, 48 h after transfection) (Figure S7D). In a Transwell assay, GPC3 siRNA-transfected cells migrated less compared to mock-transfected cells (Figure S7E), suggesting a role for GPC3 signaling in these cells. SY5Y cells also readily over-expressed transfected constructs, such as our secreted nanobodies (Figure S7F). We used our previously established *in vivo* model for neuroblastoma (Delloye-Bourgeois et al., 2017) (Figures 7C–7H and S7G). Neuroblastoma cells are engrafted within the avian pre-migratory trunk neural crest and migrate following a stereotypical ventral migratory path to the developing sympathetic ganglia and adrenal medulla. There, they express characteristic tumor features, forming tumor masses before undergoing secondary metastatic-like dissemination. Compared to embryos engrafted with scramble small interfering RNA (siRNA)-transfected SY5Y, GPC3 siRNA-transfected cells formed tumor masses almost exclusively outside the proximal and distal sympatho-adrenal (SA) territories (Figures 7C and 7D). In addition to being mistargeted, a high proportion of cells were dispersed rather than integrated in the collective migration flow. These isolated cells were either delayed within the stereotyped ventral migratory route or mislocated outside of the neural crest stream. The results suggest that interfering with the neuroblastoma source of GPC3 disrupts cell migration and targeting to the primary tumor site. Next, we over-expressed wild-type rUnc5D^lglgTSP^ and the mutant *r*Unc5D^lglgTSPGU^ in SY5Y cells prior to grafting. Both conditions resulted in an increase of isolated cells and tumor masses outside SA territories, suggesting that *r*Unc5D^lglgTSP^ has GPC3-independent functions in this system. However, the experiments also demonstrated that *r*Unc5D^lglgTSP^ WT protein promotes the formation of tumors in the most distal targets, resulting in tumor masses also below the dorsal aorta, in the developing adrenal gland and in enteric ganglia. Few individual cells were found in proximal SA derivatives (Figures 7E and 7F). In contrast, over-expression of *r*Unc5D^lglgTSPGU^ enhanced the mistargeting of tumor masses and cells out of SA territories. The results demonstrated that Unc5-GPC3 interaction directs neuroblastoma collective migration, and that the “off-target” positions we revealed by overexpressing the mutant, must be caused by other interactions. We complemented these data by over-expressing secreted Nano^break^ or Nano^glue^ (Figures 7G and 7H). Expression of Nano^break^ led to a modified migratory pattern: only a few isolated cells reached the sympatho-adrenal target derivatives, with most tumor masses formed outside. This phenotype is comparable to that found in the siRNA knockdown experiments described above. Interestingly, not only the nanobody-transfected SY5Y cells exhibited abnormal migratory and targeting patterns, but also the untransfected cells present in these grafting experiments. This suggests that Nano^break^ has both autocrine and paracrine effects on the collectivity of migrating neuroblastoma cells. Conversely, we found that grafted cells expressing Nano^glue^ had “enhanced” migratory properties, resulting in a preferential localization of tumor masses in the most distal trunk neural crest territories, similar to the over-expression of rUnc5D^IgIgTSP^. This was also reflected by the smaller number of cells that failed to reach the primary tumor site, and that these tumors were highly condensed. These results agree with the SY5Y *in vitro* stripe results presented in Figure 4K, where Nano^glue^ enhances the Unc5-GPC3 dependent cell response, while Nano^break^ reduces it. Taken together, the results show that modifying the strength of GPC3-Unc5 interaction determines cancer cell migration properties and tumor targeting in the model presented.

## Discussion

Individual receptor-ligand interactions are embedded within complicated cell surface interactomes as most receptors bind multiple ligands. A variety of complexes are formed, depending on which binding partners are available. They drive many different context-dependent signaling pathways and cellular responses. This structural/functional complexity has hampered progress with understanding where specific signaling interactions act *in vivo*. Here, we have employed an integrated structure-function approach that uses engineered mutant proteins and nanobodies to focus on the interaction between Unc5 receptors and GPC3. The structural data shows that these proteins form an unexpectedly large multimeric complex, with four copies of each molecule arranged in a pseudo-symmetric arrangement. Interestingly, multimeric extracellular complexes that go beyond simple (1:1) receptor:ligand interactions are emerging for a range of important morphogen and guidance receptors, for example, FLRT/Lphn/Teneurin (del Toro et al., 2020), netrin/RGM/neogenin (Robinson et al., 2021), Eph/ephrin (Seiradake et al., 2010, 2013), semaphorin/plexin/neuropilin (Janssen et al., 2012). This study and previous data also show how Unc5 engages in different complexes (with netrin, GPC3, FLRT, FLRT/Lphn), suggesting that a balance between different signaling configurations is dictated by the molecular composition of the environment. We found that under the harsh conditions of a native mass spectrometry experiment, the GPC3/Unc5D octamer partially disintegrates into smaller subcomponents that include 2:2 and 1:1 complexes. These presumably weaker assemblies could also have functions, perhaps at initial stages of complex formation (Figures 2A and S1F–S1H). A comparison of different Unc5 structures also reveals unexpected similarities. For example, the antiparallel packing of Unc5D in the GPC3-mediated complex is reminiscent of the arrangement in the Unc5D/Latrophilin3/FLRT2 complex, despite the different complex architectures and stoichiometries (Figures S7I and S7J). This antiparallel conformation may present a specific functional state of Unc5D; for example, it may cause distancing of the intracellular signaling domains *in cis* or allow an antiparallel *trans* interaction of Unc5D across different cells.

Post-translational glycan modifications such as C-mannosylation are emerging critical factors in receptor biology. For example, C-mannosylation and N-linked fucosylation are involved in mediating RTN4/NoGo receptor interaction with the adhesion GPCR BAI in synapse formation and neuron-glia interaction (Wang et al., 2021). The interleukin-21 receptor is stabilized by an N-linked glycan that packs against mannosylated tryptophans (Hamming et al., 2012). Enzymatic C-mannosylation of Unc5 receptors by DPY19L1 is required for effective folding and stability of Unc5 TSP domains (Shcherbakova et al., 2017, 2019). We show here that these C-mannosylated trytophans are also involved in GPC3-binding. Unc5 C-mannosylation plays a role in *C.elegans* neuroblast migration (Buettner et al., 2013), but the interacting hGPC3 N-linked glycan shown here (Figures 2K–2N) is not conserved in *C.elegans.* This suggests that the complex may not form in *C.elegans*, or assemble differently, perhaps involving Unc-40/DCC, which binds LON-2/GPC3, and affects Unc5 signaling (Blanchette et al., 2015).

Nanobodies are increasingly used to modulate protein functions, including also in clinical usage (Yang and Shah, 2020) due to their small, compact, monovalent and rigid structure, and deep tissue penetration. Here we presented Nano^glue^ and Nano^break^ to modulate Unc5-GPC3 interactions *in vitro/vivo* in monovalent form, or as tetrameric streptavidin-complexes, with enhanced binding capacities.

Neural crest-like neuroblastoma and cortical radial migration are established paradigms of cellular migration, with distinct characteristics: neuroblastoma cells undergo collective migration following a typical path through the embryonic tissue, whereas radial migration from the intermediate zone to the cortical plate relies on interactions of individual neurons with AP scaffolds. Neuroblastoma cells express varying levels of GPC3 and Unc5 receptors, with further Unc5 and GPC3 expressed in their environment. Young cortical neurons express mainly Unc5D receptors, while AP cells express GPC3 (Figures S7K–S7N). In the cortex, we find that modulating GPC3-Unc5 interaction leads to impaired radial migration. This is reminiscent of previous studies showing that any alteration to the finely balanced adhesive or repulsive forces has a detrimental impact on the migration of these neurons (Seiradake et al., 2014; del Toro et al., 2020). It is possible that the reduction of Unc5-GPC3 interactions between APs and neurons removes a repulsive force that otherwise helps the neurons detach from their scaffold as they move forward, and thereby causes migration delays. This would be consistent with studies showing that removal of other repulsive guidance factors, such as the Latrophilin-Teneurin/FLRT interaction also delays radial migration (del Toro et al., 2020). Analogous results have been obtained with other major guidance and adhesion systems, where cell migration is reduced by modulating adhesion or repulsion. Indeed, increased integrin-mediated adhesion (Haage et al., 2020) or reduced Eph-EphrinB contact repulsion reduce cell motility (Rohani et al., 2011). Inhibiting fibronectin-integrin adhesion (Ramos and DeSimone, 1996) or increasing EphB-ephrinB repulsion (Wen and Winklbauer, 2017) also impairs cell migration.

Unexpectedly, stabilizing the Unc5-GPC3 interaction artificially with Nano^glue^ reduced radial migration *in vivo*. We hypothesize that stabilizing the interaction interfered with the release mechanism from the GPC3-presenting scaffolds in neurons. This would be consistent with the lack of neural migration observed in stripe assays in the presence of Nano^glue^.

Unlike cortical neurons, many cancer cells display collective migration (te Boekhorst et al., 2016; Piacentino et al., 2020). In our neuroblastoma model, GPC3-Unc5 signaling seems to act as a switch that determines cellular cohesion. Enhancing the interaction potentiates collective migration, whereas reducing it broke the migrating cell stream up. Cancer cells can reversibly switch from collective to individual migration mode for optimal adaptation to their context (te Boekhorst et al., 2016, te Boekhorst et al., 2022). Modulation of GPC3-Unc5 interactions could thus contribute to mediating such opportunistic migration plasticity. The precise mechanism of neural crest cell targeting is poorly understood, however our results suggest that GPC3-Unc5 interaction plays a role: reducing the interaction led to the premature stopping and forming of tumors, while enhancing the interaction resulted in migration beyond typical target areas. Some cells even migrated further, along a path normally taken by the enteric neural crest to target the developing gut. In analogy with the cortical migration paradigm, we find that GPC3-Unc5 signaling must be finely balanced to achieve effective collective migration and correct targeting, possibly because it could otherwise interfere with the perception of extracellular target recognition signals.

GPC3 and Unc5 are embedded within complex protein surface interactomes. For example, Unc5 receptors bind FLRTs (Seiradake et al., 2014) and Latrophilins (Jackson et al., 2016). Given that these receptors are present in specific cortical cell populations (Seiradake et al., 2014; del Toro et al., 2020), there could be competition for the formation of different Unc5-signalling hubs. GPC3 could be interacting with receptors other than Unc5, as suggested by our *in vitro* stripe data. For example, GPC3 binds Wnts (Capurro et al., 2005) and promotes canonical Wnt/Beta-catenin signaling (Castillo et al., 2016), whose activation can provoke premature cortical migration (Woodhead et al., 2006). Wnt also regulates neural crest migration, as does Latrophilin2 in different model organisms (Becker and Wilting, 2018; Yokote et al., 2019). FLRT2 is expressed from early developmental stages in the trunk mesenchyme (Haines et al., 2006). Here, structure-guided mutants and specific functional nanobodies enabled us to focus on the Unc5-GPC3 interaction, despite the presence of other ligands. Many remaining questions could be answered using these tools; for example, regarding the roles of Unc5 and GPC3 in other tissues such as lungs, kidneys (Iglesias et al., 2008; Liu et al., 2004; Schwab et al., 2003), and the vascular system (Freitas et al., 2008; Ng et al., 2009). The Unc5-GPC3-dependent mechanisms we found in neuroblastoma migration could apply to other disseminating cancers, given that GPC3 is an oncofetal protein expressed by many pediatric solid embryonal tumors (Ortiz et al., 2019) and adult cancers (Li et al., 2018; Shimizu et al., 2019).

### Limitations of the study

Drawing conclusions across different levels of “resolution” (protein structures, cells, tissues) is challenging and relies on tools such as mutant proteins or functional nanobodies. Although these enable us to target specific protein-binding surfaces, we cannot rule out that other ligands use the same surfaces for binding and therefore could also be affected. We have mitigated this risk by targeting Unc5 and GPC3 individually in different experiments. A further limitation is that over-expression of proteins *in vivo* could lead to artifacts. We have used our mutants as controls to assess for such effects. Indeed, we found that there are interactions beyond those between Unc5-GPC3, for example in the stripe assays. We have shown that enhancing or inhibiting Unc5-GPC3 interaction impairs neuronal migration without affecting neuron morphology or differentiation. However, we have not measured the migration speed in these conditions. In the neuroblastoma model, the distribution and expression levels of endogenous and over-expressed proteins were not quantified. As above, we did not visualize the speed of migration directly, but inferred it from cell localization.

## STAR★Methods

### Key resources table

**Table:** 

REAGENT or RESOURCE	SOURCE	IDENTIFIER
**Antibodies**		

Rabbit anti-βIII Tubulin	SIGMA-Aldrich	Cat#T2200, RRID:AB_262133
Rat anti-Ctip2	Abcam	Cat#ab123449; RRID: AB_10973033
Mouse anti-Pvim	Abcam	Cat#ab20346; RRID: AB_445527
Rabbit-Satb2	Abcam	Cat#ab92446; RRID: AB_10563678
Anti-goat Alexa 594	Jackson ImmunoResearch	Cat#705-585-003; RRID: AB_2340432
Anti-human IgG	Jackson ImmunoResearch	Cat# 111-225-144, RRID:AB_2338021
Cy2 AffiniPure Goat Anti-Rabbit IgG (H + L)	Jackson ImmunoResearch	Cat# 111-225-144, RRID:AB_2338021
Goat anti-Human IgG (H + L), Alexa Fluor 594 Conjugated	Thermo Fisher Scientific	Cat#A11014 RRID:AB_1500628
Rabbit Anti-Green Fluorescent Protein (GFP) Polyclonal Antibody	Thermo Fisher Scientific	Cat# A-11122; RRID: AB_221569
Donkey anti-Rabbit IgG (H + L) Secondary Antibody, Alexa Fluor 488	Thermo Fisher Scientific	Cat # A21206; RRID: AB_2535792
Mouse Mitochondria alpha antibody	Millipore	MAB1273; RRID: AB_94052
Mouse HNK1 IgM antibody	Hybridoma Bank	Cat # 3H5; RRID: AB_2314644
Anti-mouse IgM Secondary Antibody, Alexa Fluor 647	Thermo Fisher Scientific	Cat# A21238 RRID: AB_2535807
Anti-mouse IgG, Secondary antibody, Alexa Fluor 555	Thermo Fisher Scientific	Cat # A32773 RRID: AB_2762848
Anti-HA	SIGMA-Aldrich	Cat#H3663; RRID: AB_262051
Anti-FLAG	SIGMA-Aldrich	Cat#F1804; RRID: AB_262044
Anti-6xHis Tag	Thermo Fisher Scientific	Cat#372900; RRID: AB_2533309
Anti-Penta-His antibody	QIAGEN	Cat#34660; RRID: AB_2619735
Anti-mouse Alexa 488	Abcam	Cat#ab150117; RRID: AB_2688012
Anti-rabbit Alexa 647	Abcam	Cat#ab150083; RRID: AB_2714032
Anti-mouse Cy5 secondary antibody	Abcam	Cat#ab97037; RRID: AB_10681024
Anti-rabbit Alexa 488	Abcam	Cat#ab150077; RRID: AB_150077
Anti-Mouse Cy3 secondary antibody	Abcam	Cat#ab97035; RRID: AB_10680176
Anti-Human IgG (H + L) Cross-Adsorbed Secondary Antibody, Alexa Fluor 568	Thermo Fisher Scientific	Cat# A-21090, RRID:AB_2535746
Anti-Actin antibody	Abcam	Cat# ab179467; RRID:AB_2737344
Placental alkaline phosphatase monoclonal antibody (8B6.18)	Thermo Fisher Scientific	Cat # MA5-12694; RRID: AB_10978663
Mouse anti-human IgG1-HRP	Serotec	Cat # MCA514P

**Chemicals, peptides, and recombinant proteins**		

Neurobasal medium	Invitrogen	Cat#A3582901
Penicillin Streptomycin	GIBCO	Cat#155140148
L-Glutamine	Life Technologies	Cat#25030-024
MEM Non-Essential Amino Acids solution	Life Technologies	Cat#11140035
B27 Supplement	GIBCO	Cat#17504044
Dako Mounting medium	Agilent	Cat#S3023
Fast green FCF stain	SIGMA-Aldrich	Cat#2353-45-9
Triton X-100	CarlRoth	Cat#3051
High Capacity Streptavidin Agarose beads	Thermo Fisher Scientific	Cat#20357
Streptavidin, Alexa Fluor 594 conjugate	Thermo Fisher Scientific	Cat#S11227
Probe- Mm-Unc5d-C2 RNAscope	Advanced Cell Diagnostics	Cat#480461-C2
Probe- Mm-Gpc3 - c1 RNAscope	Advanced Cell Diagnostics	Cat#418541
Dulbecco’s Modified Eagle Medium (DMEM) GlutaMAX	Thermo Fisher Scientific	Cat#31966-021
Fetal Bovine Serum (FBS)	SIGMA-Aldrich	Cat#F7524
Amphotericin B	SIGMA-Aldrich	Cat#A2942
Paraformaldehyde 32%	Electron microscopy science	Cat#15714-S
Triton 100X	Sigma-Aldrich	Cat#T9284
BSA - BSA	Sigma-Aldrich	Cat#A7906
Hoechst	Thermo Fisher Scientific	Cat# H21486
JetPrime reagent	PolyPlus - Ozyme	Cat#Pol114-15
Phosphate buffered saline, pH 7.4	Life Technologies	Cat#10010023
HEPES Free Acid 1M Solution	SIGMA-Aldrich	Cat#7365-45-9
DMEM, high glucose, pyruvate, no glutamine-500 mL	Life Technologies	Cat#21969035
IPTG	SIGMA-ALDRICH	Cat#I6758-1G
Terrific Broth	Fisher Scientific	Cat#12891660
Fetal Bovine Serum (FBS)	GIBCO	Cat#10437028
Dapi Staining Solution	Abcam	Cat#ab228549
High Capacity Streptavidin Agarose Resin	Thermo Fisher Scientific	Cat#10733315
Magnesium Sulfate, Anhydrous	SIGMA-Aldrich	Cat#746452-500G
Ampicillin Sodium Salt Biochemica	AppliChem	Cat#A0839.0025
Streptavidin from Streptomyces Avidinii	SIGMA-Aldrich	Cat#S4762-5MG
Paraformaldehyde, Powder 95%	SIGMA-Aldrich	Cat#158127-100G
Immu-mount	Thermo Fisher Scientific	Cat#10622689
Polyethylenimine (PEI)	SIGMA-Aldrich	Cat#208727
Sucrose	SIGMA-Aldrich	Cat#S0389-500G
Human IgG, Fc fragment	Jackson Immunoresearch	Cat#009-000-008-JIR
NDSB-256	Hampton Research	Cat#HR2-705
RPMI-1640 Medium	LGC Standards	Cat#ATCC 30-2001
1-Step Ultra TMB-ELISA HRP	Thermo Fisher Scientific	Cat # 34,028
Sodium deoxycholate (NaDOC)	SIGMA-Aldrich	Cat #30970
NP-40	SIGMA-Aldrich	Cat #18896
Sodium Chloride (NaCl powder)	SIGMA-Aldrich	Cat #S3014
Tris hydrochloride (Tris-HCl powder)	SIGMA-Aldrich	Cat #10812846001
Complete Protease Inhibitor Cocktail	Roche Diagnostics	Cat #04693116001

**Critical commercial assays**		

RNAscope Universal Pretreatment Kit	Advanced Cell Diagnostics	Cat#322380
RNAscope Fluorescent Multiplex Reagent Kit	Advanced Cell Diagnostics	Cat#320850
Bio-Rad protein assay	Biorad	Cat#5000001
Nucleospin RNAII kit	Macherey-Nagel	Cat#740955-10
iScript cDNA Synthesis Kit	BioRad	Cat#1708890
LightCycler480 SYBRGreen I Master1 kit	Roche Life Science	Cat#04707516001
Neon Transfection System 100 μL Kit	Thermo Fisher Scientific	Cat#MPK10025

**Deposited data**		

Mouse single-cell RNAseq data	di Bella et al. (2021)	Cat#GSE153164
Human and mouse RNAseq data (aRG, bRG and migrating neurons)	Florio et al. (2015)	Cat#GSE65000
Human Neuroblastoma tumor single-cell RNAseq data	Dong et al. (2020)	Cat#GSE137804
PDB	This study	7ZAV1
PDB	This study	7ZA2
PDB	This study	7ZA3
PDB	This study	7ZAV
PDB	This study	7ZAW

**Experimental models: Cell lines**		

Primary cortical neurons and cortex tissue from	Charles River (Maintained at the Animal Facility of Faculty of Medicine (University of Barcelona)	C57BL/6 background
SY5Y	ATCC	ATCC® CRL-2266™
IGR-N91	Laboratory of J. Bénard, Gustave Roussy Institute, Villejuif, France	Described in Ferrandis and Bénard, 1993
SHEP	Laboratory of M. Schwab, Institute for Experimental Pathology, Heidelberg, Germany.	Described in Ciccarone et al., (1989))
IMR32	ATCC	ATCC® CCL-127™
HEK293T	ATCC	CRL-3216; RRID: CVCL_0063
HEK293S	ATCC	CRL-3022; RRID: CVCL_A785
K562	ATCC	CCL-243; RRID: CVCL_0004
N2A	ATCC	ATCC® CCL-131™
WK6	ATCC	ATCC® 47,078™
C3A	ATCC	ATCC® CRL-10741™

**Experimental models: Organisms/strains**		

Embryonated eggs, naked neck strain	Elevage avicole du Grand Buisson, Saint Maurice sur Dargoire, France	N/A

**Oligonucleotides**		

ISH: GPC3-foward GCCGAAGAAGGGAACTGATTC	This study	N/A
siRNA Universal Negative Control #1	SIGMA-Aldrich	SIC001
human GPC3 siRNA; NM_004484	SIGMA-Aldrich	SASI_Hs01_00205845
PrimerPCR SYBR Green Assay: UNC5A, Human UniqueAssayID: qHsaCID0013056	Biorad	10,025,636
PrimerPCR SYBR Green Assay: UNC5B, Human UniqueAssayID: qHsaCID0021074	Biorad	10,025,636
PrimerPCR SYBR Green Assay: UNC5C, Human UniqueAssayID: qHsaCID0016268	Biorad	10,025,636
PrimerPCR SYBR Green Assay: UNC5D, Human UniqueAssayID: qHsaCED0045738	Biorad	10,025,636
PrimerPCR SYBR Green Assay: GPC3, Human UniqueAssayID: qHsaCID0016381	Biorad	10,025,636
shRNA for GPC3 knockdown: GCCGAAGAAGGGAACTGATTC	This study	N/A
Primer: Nanoglue and Nanobreak in pCAGIG and pHLSec Forward: GTAGCTGAAACCGGTCAGGTGCAGCTG GTCGAGTCTGGGG	This study	N/A
Primer: Nanoglue and Nanobreak in pCAGIG Reverse: AATTTACGTAGC GGCCGCCTAAGACAGATCCT CTTCTGAGATG	This study	N/A
Primer: Nanoglue and Nanobreak in pHLSec Reverse: GGAACCTC CGGTACCTTGGCCTCCCGGG CCGGCCGCTGGTTG	This study	N/A
Primer: hGPC3, hGPC3UG, hGPC3core, hGPC3coreUG Forward: TCTCAGG CCGAATTCATGGCCGGGACC GTGCGCACCGCGTG	This study	N/A
Primer: hGPC3 and hGPC3UG Reverse: GTGGTGCTTGGTACCTCAGTGCACCA GGAAGAAGAAGCACACC	This study	N/A
Primer: hGPC3core and hGPC3coreUG Reverse: GGAACCTCCGGTACC AACTCTACCTTTGGGCATAGACATGG	This study	N/A
Primer: hGPC3UG and hGPC3coreUG (N241Q) Forward: ggaattgaagtgatc CaGacaactgatcacctgaagttcagtaag	This study	N/A
Primer: hGPC3UG and hGPC3coreUG (N241Q) Reverse: gatcacttcaattccaagattcagag	This study	N/A
Primer: mGPC3ecto, mGPC3 488 and mGPC3core Forward: GTAGCTGAAACCG GTgacgccacctgtcaccaggtccgttc	This study	N/A
Primer: mGPC3ecto Reverse: GTGGTGCTTGGTACCggacg gcatgttccccacgctgtg	This study	N/A
Primer: mGCP3 488 Reverse: GTGGTGCTTGGTACCatc caggcttttatccagaac	This study	N/A
Primer: mGCP3core Reverse: GTGGTGCTTGGTACCtttacc cttgggcacagacatggttc	This study	N/A
Primer: mUnc5Aecto Forward: TCTC AGGCCGAATTCGCCACCATGGCTG TGCGACCTGGACTGTGGCCTGC	This study	N/A
Primer: mUnc5Aecto Reverse: GGAACCTCCGGTACCCACGTC CTCAGGGCCAGAGCTGGTGTG	This study	N/A
Primer: mUnc5AectoGU Forward: AGCTGCACCAACCCTAATCCCACC AATGGCGGCGCTTTCTGCGAG	This study	N/A
Primer: mUnc5AectoGU Reverse: AGGGTTGGTGCAGCTTCTGC TTCTCTTCTG	This study	N/A
Primer: mUnc5Becto Forward: GTAGCTGAAACCGGTtaccc atacgatgttccagattacg	This study	N/A
Primer: mUnc5Becto Reverse: GGAACCTCCGGTACCatctc ccgatgtctccagggtcagcac	This study	N/A
Primer: mUnc5BectoGU Forward: ACCTGCACCAACCCAAATCCAAC CAATGGTGGGGCCTTCTGTGAG	This study	N/A
Primer: mUnc5BectoGU Reverse: TGGGTTGGTGCAGGTTCT TGTGCGTTTCTG	This study	N/A

**Recombinant DNA**		

Plasmid: pCAGIG	Matsuda and Cepko, 2004	Cat#11159 (Addgene)
Plasmid: pCAG-miR30	Matsuda and Cepko, 2007	Cat#14758 (Addgene)
Plasmid: BLBP-GFP	Shariati et al. (2013)	Cat#63174 (Addgene
Plasmid: pCAGGS-mCherry	Gurtan et al., 2012	Cat#41583 (Addgene)
Plasmid: pHLSec	Addgene	Cat#99845
Plasmid: pADL-23c	Antibody Design Labs	SKU: PD0111

**Software and algorithms**		

Prism, version 8	Graphpad Software, USA	https://www.graphpad.com/
ImageJ (Fiji), version 1.53f51	Schneider et al., 2012	https://doi.org/10.1038/nmeth.2089
ImageJ (Fiji), version 1.53f51	Schindelin et al., 2012	https://doi.org/10.1038/nmeth.2019
CellProfiler, version 2.2.0	CellProfiler, USA	https://cellprofiler.org
RStudio, version 1.4.1106	RStudio, USA	https://www.rstudio.com/
DEP-LFQ package for R, BiocManager 1.30.16	CRAN repositories	https://bioconductor.org/packages/devel/bioc/vignettes/DEP/inst/doc/DEP.html
Seurat package for R, version 4.0.2	Satija Lab	https://satijalab.org/seurat/
Prism 9.0	GraphPad Software, USA	RRID:SCR_002798
DIALS (via XIA2)	Winter et al., 2013, 2018	https://doi.org/10.1107/S0907444913015308 https://doi.org/10.1107/S2059798317017235
CCP4 package	Winn et al., 2011	https://doi.org/10.1107/S0907444910045749
Staraniso		https://staraniso.globalphasing.org/cgi-bin/staraniso.cgic
Phenix	Liebschner et al., 2019	https://doi.org/10.1107/S2059798319011471
COOT	Emsley and Cowtan, 2004	https://doi.org/10.1107/S0907444904019158
REFMAC	Murshudov et al., 2011	https://doi.org/10.1107/S0907444911001314
CCP4i2 interface	Potterton et al., 2018	https://doi.org/10.1107/S2059798317016035
Super-Pose	Maiti et al., 2004	https://doi.org/10.1093/nar/gkh477
Privateer, MKIV version	Agirre et al., 2015	https://doi.org/10.1038/nsmb.3115.
AceDRG	Long et al., 2017	https://doi.org/10.1107/S2059798317000067
MODELLER	Webb and Sali, 2016	https://doi.org/10.1002/cpbi.3
GROMACS 2020	Abraham et al., 2015	https://doi.org/10.1016/j.softx.2015.06.001
AMBER14SB force field	Maier et al., 2015	https://doi.org/10.1021/acs.jctc.5b00255
MDAnalysis	Michaud-Agrawal et al., 2011	https://doi.org/10.1002/jcc.21787
Alpha Fold	Jumper et al., 2021; Varadi et al., 2022	https://doi.org/10.1038/s41586-021-03819-2 https://doi.org/10.1093/nar/gkab1061
VMD	Humphrey et al., 1996	https://doi.org/10.1016/0263-7855(9600018-5)
Multi-Seq VMD plugin	Roberts et al. (2006)	https://doi.org/10.1186/1471-2105-7-382
CHARMM-GUI glycan modeler	Park et al. (2019)	https://doi.org/10.1093/glycob/cwz003
BI-Aevaluation	Biacore, Cytiva	https://www.cytivalifesciences.com
Xcalibur 4.1	Thermo Fisher Scientific	https://www.thermofisher.com
MaxQuant software (Version 1.6.3.4)	Cox and Mann (2008); Cox et al. (2011)	https://doi.org/10.1093/glycob/cwz003 https://doi.org/10.1021/pr101065j

**Other**		

Series S Sensor Chip CM5	Cytiva	Cat#29149603

### Resource availability

#### Lead contact

Further information and request for resources and reagents should be directed to and will be fulfilled by the lead contact, Elena Seiradake (elena.seiradake@bioch.ox.ac.uk).

#### Materials availability

This study did not generate new unique reagents.

### Experimental model and subject details

#### Mouse embryos

All mice (C57BL/6 background) were housed with a 12h:12h light:dark cycle and food/water available *ad libitum*. All animal experiments were used in accordance with the ethical guidelines (Declaration of Helsinki and NIH, publication no. 85-23, revised 1985, European Community Guidelines, and approved by the local ethical committee (University of Barcelona, 225/17 and Generalitat de Catalunya, 404/18).

#### Chicken embryos

Naked Neck strain embryonated eggs were obtained from a local supplier (Elevage avicole du Grand Buisson, Saint Maurice sur Dargoire, France). Laying hen’s sanitary status was regularly checked by the supplier according to French laws. Eggs were housed at 18°C until use. They were then incubated at 38.5°C in a humidified incubator until the desired developmental stage, i.e, HH14 for the graft step (54 h of incubation).

#### Cell lines

HeLa, N2A, SY5Y and SY5Y:GFP (Delloye-Bourgeois et al., 2017) NB cell lines were cultured in Dulbecco’s Modified Eagle Medium (DMEM) GlutaMAX (Life Technologies). Media were each supplemented with 10% Fetal Bovine Serum (FBS), 25 U/mL Penicillin Streptomycin (Gibco), 2.5 μg/mL Amphotericin B (Sigma-Aldrich). K562 suspension cells were cultured in RPMI-1640 media supplemented with 10% FBS and 5% L-Glutamine. HEK293T and HEK293S cells were cultures in DMEM (Life Technologies) supplemented with 10% FBS, 5% L-Glutamine and 5% Non-Essential Amino Acids. Cell lines were maintained in sterile conditions in a 37°C, 5% CO2-incubator.

#### Primary cultures

Neurons were dissociated from cortices of E15.5 embryos and cultured on stripes. Neurons were cultured for 1 day *in vitro* at 37°C, 5% CO2 in Neurobasal medium supplemented with B27. Neurons were used for stripe assays and were fixed with 4% Paraformaldehyde for 10 min followed by immunostaining.

### Method details

#### Vectors and cloning

We coned constructs of human GPC3 (cDNA clone BC035972) (hGPC3, residues 1–580; hGPC3^core^, residues 32–483, UG mutant, N241Q), mouse GPC3 (Uniprot ID: Q8CFZ4) (mGPC3^ecto^, residues 31–559; mGPC3^488^, residues 31–488; mGPC3^core^, residues 31–482), mouse Unc5A (Uniprot ID: Q8K1S4) (mUnc5A^ecto^, residues 1–359), mouse Unc5B (Uniprot ID: Q8K1S3) (mUnc5B, residues 26–934; mUnc5B^ecto^, residues 26–362), into the Age1-Kpn1 or EcoR1-Kpn1 cloning site of vectors from the pHLSec family (Aricescu et al., 2006). For protein purification, we used pHLSec vectors which also code for a C-terminal 6xHis-tag, for SPR we used a C-terminal Avi-tag. We used previously published rUnc5D constructs and derivatives thereof as indicated in the text (Jackson et al., 2015, 2016; Seiradake et al., 2014; del Toro et al., 2020), including (rUnc5D, rUnc5D^ecto^, rUnc5D^IgIgTSP^, rUnc5D^IgIg^, rUnc5D^TSPTSP^, FU mutant (W85N + S87T), human Unc5B (Q8IZJ1) (hUnc5B^ecto^), hUnc5A (Uniprot ID: Q6ZN44) (hUnc5A^ecto^, equivalent to Unc5A^Ig12T1^), mouse FLRT2 (FLRT2^LRR^). For cell binding and functional assays, full length constructs were used, either cloned into a pHLSec vector that encodes an intracellular mVenus, or the pCAGIG vector (Addgene; Matsuda and Cepko, 2004), for visualisation. pCAGIG was modified to express mCherry instead of GFP, for certain experiments, and is then referred to as pCAGIC. For the nanobody expression and purification in WK6, Nano^glue^ and Nano^break^ in pADL-23c vector were used. Nanobodies were cloned into pHLsec C-terminal Avi-tag, for biotinylation in HEK293T cells. They were subcloned in the pCAGIG vector with the pHLSec-derived secretion signal, for *in vivo* experiments. For the ELISA experiments, human Unc5D (residues 33–379, Uniprot ID: Q6UXZ4) and human GPC3 (residues 25–563, Uniprot ID: P51654) were cloned into a modified pCMV6-XL4 vector in frame with an N-terminal FLAG and C-terminal human Fc or alkaline phosphatase fragments.

#### ELISA protocol

An ELISA-based assay was used to identify novel ligands for Unc5D-AP. The experiment was done in duplicate. Twenty μL of a solution at 3 μg/mL of mouse anti-AP in 1X PBS was added to each well of 96-well plates using an automated multichannel pipette (Viaflo Assist, Integra), sealed and incubated overnight at 4C. The following day, the plate was washed once with PBS and 1% casein was added as a blocking agent, which was removed after 1 h at room temperature using an automated microplate washer (HydroSpeed, Tecan). Next, to each well, 20 μL of Unc5D-AP conditioned medium, containing 2 μL of monoclonal mouse anti-human IgG1-HRP was added using an automated plate copier (Viaflo96, Integra) along with 20 μL of culture medium from 95 different ecto-Fc prey proteins. Plates were sealed and incubated for 4 h at room temperature in the dark. Plates were subsequently washed, and 35 μL 1-Step Ultra TMB-ELISA HRP substrate was added using an automated multichannel pipette (Viaflo Assist, Integra); after 1 h incubation at room temperature, the absorbance at 650 nm was recorded with a microplate Spectramax i3 plate reader (Molecular Devices). Finally, plates were scanned to obtain matching images of the 650 nm reading. A positive control (known interactors NRXN/NLGN1) was used (Ozgul et al., 2019).

#### Protein expression and purification

Recombinant protein expression and purification were performed as described (Aricescu et al., 2006; Seiradake et al., 2015). Briefly, adherent HEK293T or GnTI-deficient HEK293S cells were transiently transfected with the relevant plasmids using polyethylenimine (PEI) and grown for 5–10 days. Cell culture media were filtered to remove dead cells and buffer-exchanged to phosphate buffer saline (PBS) containing also 250 mM NaCl and 20 mM Tris (pH 7.5). Conditioned media were passed through HisTrap HP columns (GE Healthcare), washed with buffer supplemented with 40 mM imidazole and bound proteins were eluted using 20 mM Tris pH 7.5, 300 mM NaCl and 500 mM imidazole. The eluate was then subjected to size exclusion chromatography using Superdex 200 16/60 (GE Healthcare) in 10 mM Tris-HCl (pH 7.5) and 200 mM NaCl.

Nanobodies were expressed in *E. coli* strain WK6., grown at 37°C in Terrific Broth until an OD600 = 0.8. Expression was induced with 150 μM IPTG and incubation at 21°C for 16 h. Cells were harvested by centrifugation (6,000xg, 15 min). Cell pellets were resuspended and incubated in ice-cold 20% sucrose, 30 mM Tris-HCl, pH 7.5, 2 mM EDTA buffer for 20 min. The cell suspension was clarified by centrifugation (10.000 rpm, 20 min, 4°C). After the supernatant collection, cell pellets were resuspended and incubated in ice-cold 30 mM Tris- HCl, pH 7.5, 5 mM MgSO4 buffer for 20 min. The cell suspension was clarified by centrifugation (10.000 rpm, 20 min, 4°C). The supernatant was filtered, supplemented with 150 mM NaCl and 2 mM Imidazole and passed through HisTrap HP columns. The column was washed with 200 mL wash buffer (30 mM Tris-HCL, pH 7.5, 150 mM NaCl, 5 mM Imidazole) and the protein was eluted in elution buffer (30 mM Tris-HCL, pH 7.5, 150 mM NaCl, 500 mM Imidazole). Elution was loaded onto a Superdex200 16/600 HiLoad column in 20 mM Tris-HCL, pH 7.5, 200 mM NaCl.

For protein biotinylation in HEK293 cells, protein constructs in pHLsec C-terminal Avi-tag were co transfected with a vector encoding BirA (biotin ligase). Cell culture medium was supplemented with 100mM biotin. Proteins were purified as previously, but in ice-cold conditions. For the nanobody-streptavidin complexes, streptavidin (Streptavidin-Alexa Fluor 594 conjugate, Thermo Fisher Scientific, or Streptavidin from *Streptomyces avidinii*, Sigma-Aldrich) was mixed with an excess of biotinylated nanobodies, incubated overnight at 4°C and subjected to size exclusion chromatography using Superdex 200 16/60 (GE Healthcare) in 20 mM Tris-HCl (pH 7.5) and 200 mM NaCl.

#### Protein X-Ray crystallography

Proteins that were expressed in GnTI-deficient HEK293S cells were used for crystallisation trials. Crystals were grown by the vapor diffusion method at 18°C by mixing the protein solution and crystallization solution in a 1:1 ratio. Purified hGPC3^core^ was concentrated to 5.3 mg/mL, and crystals were obtained using crystallization solution 1 (20% ethylene glycol, 10% w/v PEG 8000, 0.1 M Tris/BICINE (pH 8.5) and 0.02 M of amino acids (L-Na-glutamate, alanine, glycine, lysine-HCl, and serine)). Crystals of mGPC3^core^ were obtained by concentrating the protein to 9.9 mg/mL in the presence of 100 mM NDSB256 and using crystallization solution 2 (0.2 M ammonium nitrate and 20% w/v PEG3350). Crystals of the complex hGPC3^core^ and rUnc5D^IgIgTSP^ were obtained by mixing the two proteins in a 1:1 M ratio and concentrating them to 5.8 mg/mL. The protein solution was then mixed with crystallisation solution 1. Crystals of the mGPC3^488^ and rUnc5D^IgIgTSP^ complex were obtained by mixing the two proteins in a 1:1 M ratio, concentrating to 7.1 mg/mL and mixing with 15% w/v PEG 3000, 20% v/v 1, 2, 4-butanetrol, 1% w/v NDSB 256, 0.1 M Gly-Gly/AMPD (pH 8.5) and 0.2 M of amino acids (DL-arginine HCl, DL-threonine, DL-histidine HCl H_2_O, DL-5-hydroxylysine HCl, *trans*-4-hydroxyl-L-proline).

#### Structure determination

Crystals of hGPC3^core^, hGPC3^core^/rUnc5D^IgIgTSP^ and mGPC3^core^/rUnc5D^IgIgTSP^ were flash-cooled in their original crystallization condition. Crystals of mGPC3^core^ were cryoprotected by adding 20% v/v glycerol to the original crystallization solution. All diffraction data were collected at the Diamond Light Source synchrotron at 100K. Data was integrated using DIALS (via XIA2)(Winter et al., 2013, Winter et al., 2018), and integrated intensities were merged and scaled using programs from the CCP4 package (Winn et al., 2011). The data of hGPC3^core^/rUnc5D^IgIgTSP^ was also processed with Staraniso (Vonrhein et al., 2018). The structures were solved by molecular replacement (MR) using the models of h/mGPC3^core^, rUnc5D^IgIgTSP^ (Jackson et al., 2016) and PHASER (McCoy et al., 2007). Initial phases of mGPC3^core^ were obtained by MR using the central lobe of GPC1 and DLP structures (Awad et al., 2015; Kim et al., 2011). We performed iterative cycles of model building and refinement in Phenix (Liebschner et al., 2019). Manual model building was performed in Coot (Emsley and Cowtan, 2004), and models were all atom refined using REFMAC (Murshudov et al., 2011) and Phenix (Liebschner et al., 2019). For the complexes, we used high-resolution models of individual components as targets, non-crystallographic symmetry (NCS), TLS and secondary structure restraints. The quality of the final models was assessed by MolProbity (Davis et al., 2007) and the CCP4i2 validation task (Potterton et al., 2018). Superpositions were done with Super-Pose (Maiti et al., 2004).

#### Glycan modeling, refinement and validation

N-glycans were built into positive omit electron density using the Coot N-linked carbohydrate building tool (Emsley and Crispin, 2018), then corrected manually where obvious discrepancies between map and model were encountered. The mannosylated tryptophans showed extra omit density consistent with this modification, which has been recently shown to force the mannoside moiety into a ^1^C_4_ conformation (an inverted chair) to keep the alpha linkage in a clash-avoiding equatorial conformation. In order to increase the observation to parameter ratio and restrain the mannoside’s ring conformation individually, external restraints for both N- and C-glycosylation were generated using the MKIV version of the Privateer software (Agirre et al., 2015). Restraints for the MAN-TRP covalent linkages were created using the AceDRG software (Long et al., 2017) through its CCP4i2 interface (Potterton et al., 2018). Glycans were iteratively refined using the REFMAC5 software and validated by Privateer software (Agirre et al., 2015).

#### Molecular dynamics simulations and modeling

To simulate the hGPC3-rUnc5D complex, we followed essentially the same protocol as previously described to refine the structures of X-ray crystallography-derived complexes (Jackson et al., 2016; del Toro et al., 2020). Missing residues were modeled using MODELLER (Webb and Sali, 2016). As the purpose of this simulation was to identify protein-protein interactions, we removed the glycan parts. The proteins were solvated in TIP3P water with 150 mM NaCl. Molecular dynamics simulations were performed using GROMACS 2020 (Abraham et al., 2015) with the AMBER14SB force field (Maier et al., 2015). The system was first energy minimized and then equilibrated following a two steps procedure of constant temperature followed by constant pressure equilibrations (Lemkul, 2019). The 500 ns of production were run at 310 K and 1 bar in an NPT ensemble, using the velocity-rescaling thermostat (Bussi et al., 2007) coupled with the Parrinello–Rahman barostat (Parrinello and Rahman, 1981). To focus only on residue side chains movements, we keep the proteins backbone constrained while side chains were allowed to move freely during the course of the simulation. We used MDAnalysis (Michaud-Agrawal et al., 2011) to perform hydrogen bond analysis as previously described (del Toro et al., 2020). The Jupyter notebook used to perform such an analysis is available at: https://github.com/MChavent/Hbond-analysis. Briefly, we used a donor-acceptor distance cut-off of 3.0 Å and a cut-off angle of 120°. The hydrogen bond stability was defined as the percentage of the simulated time in which a residue forms stable hydrogen-bonds with its partner.

The models of membrane-bound GPC3 and Unc5D (Figures S7L and S7M) were produced using structures presented here, and Alpha-fold models of the hGPC3 C-terminal region and the rUnc5D TSP2 and transmembrane domains (Jumper et al., 2021; Varadi et al., 2022). To position the Unc5D TSP2 domain, we ran 100 ns of atomistic simulations on the Alpha-fold model containing Ig2+TSP1+TSP2 (as described above) without constraints. While the Ig1 -TSP1 linkage was stable in these simulations, we observed that the TSP2 domain explored a range of positions relative to TSP1, possibly due to the presence of a proline residue in the linker between TSP1 and TSP2. We extracted 7 representative structures from the molecular dynamics trajectory, shown superposed in Figure S7K. We used the Multi-Seq VMD plugin (Roberts et al., 2006) and VMD (Humphrey et al., 1996) to superimpose via the TSP1 domains for this figure. Missing linkers for Unc5D and GPC3 were added using MODELLER (Webb and Sali, 2016). We added heparan sulfate glycans and GPI anchors using the CHARMM-GUI glycan modeller (Park et al., 2019).

#### SPR

Equilibrium binding experiments were performed at 25°C using a Biacore T200 instrument (GE Healthcare) using PBS +0.005% (v/v) polysorbate 20 (pH 7.5) or 20mM Tris +200mM NaCl +0.005% (v/v) polysorbate 20 (pH 7.5) as running buffers. Glypican, Unc5 or nanobody constructs were biotinylated enzymatically at a C-terminal Avi-Tag and coupled to a streptavidin-coated CM5 chip. Protein analytes were injected over the chip in 2-fold dilution series for 500 s, followed by at least 25 s dissociation time. The regeneration buffer used was 2 M MgCl_2_. Data were analyzed using the BI-Aevaluation software. Indicative K_D_ and R_max_ values were obtained by nonlinear curve fitting of a 1:1 Langmuir interaction model (bound = (R_max_ x C)/(K_D_ + C), where C is analyte concentration calculated as monomer.

#### Cell binding assay

HEK293T cells grown on coverslips were transfected using mVenus-tagged construct with 3 μg of DNA and 9 μL of PEI. Eighteen hours after transfections, cells were incubated with buffer (HBSS with 1% BSA and 10 mM HEPES (pH 7.5)) for 30 min on ice, and then with buffer containing 0.5 μg purified His-tagged protein per coverslip that was previously pre-clustered (20 min at room temperature) with anti-His (mouse; Thermo Fisher Scientific) in a 1:2 (protein:antibody) ratio for 60 min on ice. Cells were then washed with PBS and fixed with 4% PFA for 20 min, and then washed using PBS supplemented with 50 mM ammonium chloride. Cells were then incubated in the dark with anti-mouse-Cy3 in a 1:7.5 (protein:antibody) ratio in buffer for 60 min on ice. The cells were washed with PBS, stained with DAPI (0.1 μg/mL) and mounted using Immu-Mount. Imaging for data analysis was done with a Nikon ECLIPSE TE2000-U inverted fluorescence microscope. Analysis was performed in ImageJ/Fiji (Schindelin et al., 2012; Schneider et al., 2012) using the co-localisation tool. The co-localisation area of red pixels (soluble protein) with green pixels (cell-bound protein) was normalised against the area of cell-bound protein and converted into a percentage. For the statistical analysis we used a one-way ANOVA test, with a Tukey’s post-hoc test in Graphpad Prism (version 9 for MacOS, GraphPad Software, San Diego, California USA, https://www.graphpad.com). Significance was determined when p < 0.5.

#### Native mass spectrometry experiments

Unliganded mGPC3^core^ and rUnc5D^IgIgTSP^ were concentrated separately to 10 μM, dialyzed against 1M ammonium acetate buffer (pH 7.5) overnight at 4°C, and injected at 3 μM concentration. The mGPC3^core(R355A/R358A)^ - rUnc5D^IgIgTSP^ complex was concentrated to 15 μM (assuming a 4:4 stoichiometry) and dialyzed against 200mM ammonium acetate (pH 7.5) and injected at 3.3 μM concentration. The protein samples were loaded into in-house prepared gold-coated capillary needles (Harvard Apparatus) and were injected directly to the mass spectrometer. The experiments were performed using a Q-Exactive UHMR Hybrid Quadrupole-Orbitrap mass spectrometer (Thermo Fisher). Typically, 3 μL of protein solution was electrosprayed from gold-coated capillaries. The instrument parameters for MS are as follows: 1.2 kV capillary voltage, S-lens RF 200%, quadrupole selection from 1,500 to 20,000 *m*/*z* range, in-source trapping energy (0-20V), nitrogen UHV pressure of 6.07 × 10^−10^ mbar and capillary temperature of 100 °C. The resolution of the instrument was 17,500 at *m*/*z* = 200 (transient time of 64 ms). The noise level was set at 3 rather than the default value of 4.64. For MS/MS analysis, collisional activation in the HCD cell was provided (0–300 V). Calibration of the instruments was performed using a 10 mg/mL  solution of cesium iodide in water. Data were analyzed using the Xcalibur 4.1 (Thermo Scientific).

#### LC-MS/MS identification of tryptophan mannosylation

Tryptophan mannosylation was verified by LC-MS/MS. 5 μg of each protein (1 mg/mL) was diluted to a final volume of 100 μL in denaturing buffer (8 M Urea, 50 mM Ammonium Bicarbonate). The sample was reduced with DTT (2 μL of 200 mM solution in denaturing buffer) at 56°C for 25 min, followed by alkylation with iodoacetamide (4 μL of 200 mM solution in denaturing buffer) at room temperature for 30 min. Alkylation was quenched by further addition of 2 μL of DTT solution. The samples were further diluted 3-folds using 50 mM Ammonium Acetate buffer. Trypsin was added to the sample in 1:50 (Enzyme: Protein (w/w)) ratio. The sample was incubated at 37°C for 16 h. Next day, the digested sample was divided into two fractions. One of the fraction was quenched using 10% Formic acid solution. To the other fraction, AspN was added in 1:20 (Enzyme: Protein (w/w)) ratio. The sample was incubated further at 37°C for 4 h after which it was quenched using 10% Formic acid solution. Resulting peptides were analyzed on an UltiMate 3000 UHPLC system (Thermo Fisher) connected to an Orbitrap Eclipse Tribrid mass spectrometer (Thermo Fisher). The peptides were trapped on an guard column (Acclaim PepMap 100, 75 μm × 2 cm, nano viper, C18, 3 μm, 100 Å, Thermo Fisher) using solvent A (0.1% Formic acid, water). The peptides were separated on an Acclaim PepMap analytical column (75 μm × 150 mm, RSLC C18, 3 μm, 100 Å) using a linear gradient (length: 90 min, 6%–45% solvent B (0.1% formic acid, 80% acetonitrile, 20% water), flow rate: 300 nL/min). The separated peptides were electrosprayed directly into the mass spectrometer in the positive ion mode using data-dependent acquisition with a 3 s cycle time. Precursors and products were detected in the Orbitrap analyzer at a resolving power of 60,000 and 30,000 (@ *m*/*z* 200), respectively. Precursor signals with an intensity >1.0 x 10^−4^ and charge state between 2 and 7 were isolated with the quadrupole using a 0.7 *m*/*z* isolation window (0.5 *m*/*z* offset) and subjected to MS/MS fragmentation using higher-energy collision induced dissociation (30% relative fragmentation energy). MS/MS scans were collected at an AGC setting of 1.0 x 10^4^ or a maximum fill time of 100 ms and precursors within 10 ppm were dynamically excluded for 30 s.

Raw data files were processed using MaxQuant software (Version 1.6.3.4), having in-built Andromeda search engine (Cox and Mann, 2008; Cox et al., 2011). The peak lists were searched against individual Unc and common contaminant proteins. Carbamidomethylation was kept as fixed modification whereas acetylation (protein N-term), oxidation (methionine), and hexose (tryptophan) were used as variable modifications. Protein and peptide false discovery rate was kept at 1%. Trypsin and AspN were set as the protease and up to four missed cleavages were allowed.

#### Nanobody generation

Antibodies to hGPC3^core^ were raised in a llama by intra-muscular immunization with purified protein using Gerbu LQ#3000 as the adjuvant. Immunisations and handling of the llama were performed under the authority of the project license PA1FB163A (University of Reading, UK). Total RNA was extracted from peripheral blood mononuclear cells, and VHH complementary DNAs were generated by RT-PCR. The pool of VHH-encoding sequences was amplified by two rounds of nested PCR and cloned into the SfiI sites of the phagemid vector pADL-23c as previously described (Huo et al., 2021). Electrocompetent *E. coli* TG1 cells were transformed with the recombinant pADL-23c vector, and the resulting TG1 library stock was infected with M13K07 helper phage to obtain a library of VHH-presenting phages. Phages displaying VHHs specific for hGPC3^core^ were enriched via two rounds of bio-panning on biotinylated hGPC3^core^, and individual phagemid clones were picked. VHH-displaying phages were recovered by infection with M13K07 helper phage and tested for binding to hGPC3^core^ by enzyme-linked immunosorbent assay (ELISA). Phage binders were ranked according to the ELISA signal and grouped by CDR3 sequence identity.

#### Cell aggregation assay

K562 suspension cells were cultured in RPMI-1640 media supplemented with 10% FBS and 5% L-Glutamine. The cells were harvested by a 3 min spin at 200g, washed with PBS, spinned again and resuspended in R buffer (Neon transfection system). Cells at a concentration of 2x10^7^ cells/ml were transfected with control pCAGIG/gCAGIC plasmids, or those coding for Unc5 or GPC3 constructs using the Neon transfection system for electroporation (Settings: 1450V, 3 pulses, 10 ms). Twenty-four hours after transfection, cells were harvested, passed through a 40 μm cell-strainer and used at a concentration of either 2 x 10^5^ cells/ml or 4 x 10^5^ cells/ml in aggregation media (Neurobasal-A media supplemented with 2 mM L-glutamine, 10% FBS, 4% B-27 and 20 mM HEPES). For the competition experiments, different amounts of nanobodies (5, 10 and 50 μg) were added at this stage. Cells were then left to aggregate at 37°C, 5% CO_2_ and 250 rpm for 90 min. After the incubation, cells were diluted in 2 mL of PBS and imaged in a 6-well plate using a Nikon ECLIPSE TE2000-U inverted fluorescence microscope. Images presented were obtained using Inverted DeltaVision widefield microscope at 37°C. The total area of cells and the total area of the aggregates for each picture were calculated using the Analyze particle tool in Fiji (Schindelin et al., 2012). The threshold used to distinguish cells and aggregates was determined at 1284 μm^2^ (>3/4 cells). For the statistical analysis we used a one-way ANOVA test, with a Tukey’s post-hoc test in GraphPad Prism (version 9 for MacOS, GraphPad Software, San Diego, California USA, https://www.graphpad.com). Significance was determined when p < 0.5.

#### K562 protein expression tests

For surface staining, K562 cells were harvested 24 h after electroporation and cooled to 4°C. The cells were then incubated with blocking buffer: HBSS with 1% BSA and 10 mM HEPES (pH 7.5) for 30 min. For Unc5D expressing cells, cells were incubated with anti-HA (mouse; Sigma-Aldrich) antibody that was pre-clustered with secondary antibody-Cy5 for 40 min (ratio 1:7.5 for primary:secondary antibody). For GPC3 expressing cells, cells were incubated anti-FLAG (rabbit; Sigma-Aldrich) antibody that was pre-clustered with secondary antibody conjugated with Alexa 647 for 40 min (1:7.5 ratio). Cells were washed with PBS, fixed with 4% PFA, DAPI-stained, washed and resuspended in 30% sucrose/PBS. Cells were then deposited onto microscope slides using a homemade cytospin (Sisino et al., 2006). Imaging was performed using an Inverted DeltaVision widefield microscope with CCD. Colocalization for the surface quantification was performed using ImageJ/Fiji (Schindelin et al., 2012; Schneider et al., 2012) as previously stated. For the total amount of protein, K562 cells expressing the desired constructs were harvested 24 h after electroporation, lysed by sonication (2 pulses of 5 s), and analyzed on SDS-page/western blot with mouse anti-His, rabbit anti-FLAG, mouse anti-HA or mouse anti-Actin. For the statistical analysis we used a one-way ANOVA test, with a Tukey’s post-hoc test in GraphPad Prism (version 9 for MacOS, GraphPad Software, San Diego, California USA, https://www.graphpad.com). Significance was determined when p < 0.5.

#### Endogenous protein expression tests in cell lines

Whole cell extract was isolated using RIPA buffer (150mM NaCl, 50 mM Tris-HCL pH7.35, 1% DOC, 1% NP40) supplemented with protein inhibitor (Roche Diagnostics). The concentration of isolated proteins was determined using Bradford assays (Bio-Rad). Western Blot analysis was performed using the following primary antibodies: anti-GPC3 (1/1000; Thermo Fisher Scientific), anti-Unc5D (1/1000, R&D systems), anti-g-tubulin (1/1000, Sigma-Aldrich). Anti-mouse IgG HRP (1/10,000, Sigma-Aldrich) and anti-goat IgG HRP (1/10,000, Sigma-Aldrich) were used as secondary antibodies.

#### Analysis of published single cell RNASeq dataset

Single Cell RNASeq of human neuroblastoma tumor samples were exploited from Dong et al. (2020); GEO ID: GSE137804) public dataset. Raw sequencing data were processed following the partially published method details, explaining the different UMAP obtained in the present study. The R package Seurat (v4.0.1) was used to calculate the quality control metrics. To filter out low quality cells, we kept all cells with at least 200 detected genes and less than 10% of mitochondrial genes. Doublet cells were removed with the R package DoubletFinder (v2.0.3). To merge all samples without biasing the analysis with batch effects, while preserving the biological variation, we applied Seurat integration and re-computed a clustering based on the corrected matrix. Single RNAseq data for cortex samples were obtained from the published NCBI Gene Expression Omnibus with accession numbers GSE65000 (Florio et al., 2015)and GSE153164 (di Bella et al., 2021). We used the same UMAP coordinates and metadata information with the cluster categorization provided by the authors.

#### Plasmids, siRNAs, cell transfection for grafting

Control siRNA (siRNA scr) (siRNA Universal Negative Control #1 SIC001) and human GPC3 siRNA (NM_004484; SASI_Hs01_00205845) were purchased from Sigma-Aldrich and used at a concentration of 50 nM pCAGIG vectors encoding for nanobodies or Unc5D^IgIgTSP^ constructs were used at a concentration of 2 μg/mL. For siRNA and plasmids transfection, cells were transfected with JetPrime according to the manufacturer’s guidelines (PolyPlus).

#### RNA isolation and quantitative real-time PCR (qRT-PCR)

For qRT-PCR analysis, total RNA was extracted from cells using the Nucleospin RNAII kit (Macherey-Nagel). One μg of total RNA was reverse-transcribed using the iScript cDNA Synthesis Kit (BioRad). qRT-PCR was performed using the LightCycler480 SYBRGreen I Master1 kit (Roche Life Science) and the CFX Connect Real-Time PCR Detection System (BioRad). The following list of primers was used in the study:

Human HPRT: Fwd: for TGACACTGGCAAAACAATGCA/Rev: GGTCCTTTTCACCAGCAAGCT;
Human UNC5A: PrimerPCR SYBR Green Assay; qHsaCID0013056
Human UNC5B: PrimerPCR SYBR Green Assay; qHsaCID0021074
Human UNC5C: PrimerPCR SYBR Green Assay; qHsaCID0016268
Human UNC5D: PrimerPCR SYBR Green Assay; qHsaCED0045738
Human GPC3: PrimerPCR SYBR Green Assay; qHsaCID0016381

#### Immunofluorescence on chick embryo slices

Chick embryos of interest were harvested and fixed in 4% paraformaldehyde (PFA). Embryos were embedded in 7.5% gelatin and 15% sucrose in PBS to perform 20 μm transverse cryosections. Permeabilization and saturation of sections were performed in PBS with 3% BSA and 0.5% Triton. The following primary antibodies were applied to sections: anti-HNK1 mouse IgM (1/50, 3H5, DSHB), anti-GFP rabbit IgG (1/500, Thermo Fisher Scientific), anti-mitochondria mouse IgG (1/500, Millipore) and the following secondary antibodies: Alexa 647 anti-mouse IgM (1/500, Thermo Fisher Scientific); Alexa 488 anti-rabbit IgG (1/500, Thermo Fisher Scientific), Alexa 555 anti-mouse IgG (1/500, Thermo Fisher Scientific) and. Nuclei were stained with Hoechst (Thermo Fisher Scientific). Slices were imaged with a confocal microscope (Olympus, FV1000, X81) using a 10× objective. The position of isolated cells and tumor masses was pointed on a reference image for each slice in which tumor cells could be detected, using Fiji (Schindelin et al., 2012).

#### Transwell migration assays

Transfected SY5Y cells were plated on the porous filter of the upper chamber of transwell culture dishes (8 μm pore size; BD Falcon, NJ, 5 x 10^4^ cells/ml). The cells were then incubated for 60 h in a 37°C, 5% CO2-incubator. Cells retained on the upper face of the membrane were scrubbed using cotton swabs. The transwell culture dishes were then fixed with with 4% PFA for 30 min, before washing with 3 successive PBS-baths and mounting in Mowiol. Migrating cells were counted using a confocal microscope (Olympus, FV1000, X81).

#### Stripe assays

We prepared the stripe assays essentially as previously described (del Toro et al., 2020).

50 μg/mL of Fc recombinant protein, GPC3^core^ or GPC3^coreUG^, were mixed with Alexa 594-conjugated anti-hFc antibody (Thermo Fisher Scientific) in PBS. Proteins were injected into matrices (90 μm width) (17,546,017) and placed on 60 mm dishes, resulting in red fluorescent stripes. After 30 min incubation at 37°C, dishes were washed with PBS and matrices removed. Dishes were coated with 50 μg/mL Fc or GPC3^coreUG^ protein mixed with 120 μg/mL anti-hFc (Jackson ImmunoResearch) for 30 min at 37°C and washed with PBS. Stripes were further coated with 20 μg/mL Laminin in PBS for at least 2 h and washed with PBS. Cortical neurons (E15.5) were cultured on the stripes in Neurobasal medium supplemented with B27 (Gibco). For testing the effects of nanobodies, neurons were cultured in medium containing 50 μg/mL streptavidin Alexa 594 (CN), Strep-Nano^break^ or Strep-Nano^glue^. After 24 h neurons were fixed with 4% PFA in PBS for 20 min at room temperature (RT). Neurons were washed and incubated with rabbit monoclonal anti-beta-III tubulin antibody (Sigma-Aldrich) after 20 min permeabilization in 1% BSA, 0.1% Triton X-100 in PBS. Cy2 anti-rabbit IgG secondary antibody (Jackson ImmunoResearch, cat#111-225-144) was used to visualize the tubulin signal. Nuclei were counterstained with DAPI before mounting. The numbers of beta-III-tubulin-positive (green) pixels on red or black stripes were quantified with ImageJ (version 1.53f) (Schneider et al., 2012) using a custom-made automatic macro that is available upon request.

For cell lines, 50 μg/mL of GPC3^core^ protein was mixed with Alexa 568-conjugated anti-hIgG antibody (Thermo Fisher Scientific) in PBS. Protein was injected into matrices (90 μm width) (17,546,017) and placed on 60 mm dishes, resulting in red fluorescent stripes. After 30 min incubation at 37°C, dishes were washed with PBS and matrices removed. Dishes were coated with 50 μg/mL GPC3^coreUG^ protein mixed with 120 μg/mL anti-hFc (Jackson) for 30 min at 37°C and washed with PBS. HeLa, SY5Y or N2A cells were cultured on the stripes in 2% FBS medium, with 50 μg/mL Strep-Nano^break^ or Strep-Nano^glue^, or without nanobody (control). After 16 h cells were fixed with 4% PFA in PBS for 20 min at room temperature and nuclei were counterstained with DAPI before mounting and imaging. The number of DAPI positive pixels on red or black stripes was quantified.

#### RNA In situ hybridization (ISH) and Immunohistochemistry

Embryonic brains were fixed in 4% PFA overnight. 10 μm Cryo-sections were pre-treated using the RNAscope Universal Pretreatment Kit (Advanced Cell Diagnostics). RNA *In Situ* Hybridizations (ISH) were performed using the RNAscope Fluorescent Multiplex Reagent Kit (Advanced Cell Diagnostics) according to manufacturer’s instructions. The target genes (Unc5D and GPC3) are listed in the Key resources table. Sections were immunostained using mouse anti-Pvim ⅓00 (Abcam), rat anti-Ctip2 1/600 (Abcam) or rabbit anti-Satb2 (Abcam) in combination with the Alexa Fluor 488-, 555-, and 647-conjugated mouse/rat/rabbit secondary antibodies (Abcam; 1/400). Both primary and secondary antibodies were diluted in 2% BSA, 0.3% Triton X-100, PBS. Nuclei were counterstained with DAPI before mounting. Images were acquired using a Zeiss LSM880 confocal laser scanning microscope using a 20x, 40× objective and 2 Airy disk pinhole, and processed with ImageJ software.

#### In utero electroporation

*In utero* electroporation was performed at E13.5 with anesthetized C57BL/6 mice as previously described (del Toro et al., 2020). DNA plasmids were used at 2 μg/μL and mixed with 1% fast green (Sigma-Aldrich, final concentration 0.2%). Plasmids were injected into the ventricle with a pump-controlled micropipette. After injection, six 50 ms electric pulses were generated with electrodes confronting the uterus above the ventricle. The abdominal wall and skin were sewed, and the mice were kept until E16.5 embryonic stage.

To knockdown GPC3 by shRNA *in vivo*, we used the best out of 3 different tested target regions embedded in the in the pCAG-miR30 vector, with the following sequence: GCCGAAGAAGGGAACTGATTC. This shRNA was validated in HEK293T cells, by co-transfection with GPC3 followed by western blotting. pCAGGS-mCherry (Gurtan et al., 2012) was used to visualise electroporated cells. Secreted versions of Unc5D extracellular domains (Unc5DIgIgTSP and Unc5DIgIgTSPGU) as well as the nanobodies (Nanoglue and Nanobreak) were cloned into the pCAGIG vector. The expression of all constructs was validated by expression in HEK293T cells and analyzed on western blots.

#### Pull-down experiments

Pull-down experiments were performed as previously described (del Toro et al., 2020). Fresh E15.5 mouse cortices were homogenized for 1 min at 4°C with an electric homogenizer using the following lysis buffer: 50 mM Tris-HCl (pH 7.4), 150mM NaCl, 2mM EDTA, 1% Triton X-100 and protease inhibitors (Sigma-Aldrich 04,693,132,001). Samples were incubated on ice for 20 min and centrifuged for 10 min at 3000 rpm. Supernatant was collected and protein was measured using the Bio-Rad protein assay (Biorad, 5,000,001). 1.5 mg of protein at a final concentration of 2 μg/μL in lysis buffer (volume: 750 μL) was used for each pull-down. Control pull-down contained lysate and 40ul of high-capacity streptavidin agarose resin (Thermo Fisher Scientific, 20,357, 50% w/v), whereas the nanobody condition contained the same beads plus 2μg of biotinylated Nano^glue^. Samples were incubated overnight at 4°C under rotatory agitation. The next day, agarose beads were centrifuged for 5 min at 3000 rpm and washed three times (first wash with 400 μL of lysis buffer, second wash with 1:1 (v/v) lysis buffer:PBS, last wash only PBS). Pulled-down samples were processed for mass spectrometry (MaxQuant run, Proteomic facility, Max Planck Institute of Biochemistry, Martinsried, Germany). Volcano plots were generated using the DEP package in R-studio.

Pull-down experiments to investigate the effects of Nano^glue^ and Nano^break^ on the Unc5 – GPC3 interaction were performed by coupling biotinylated hGPC3^core^ to high-capacity streptavidin agarose resin (Thermo Fisher Scientific, 20,357) after incubation of the beads with the biotinylated protein for 1 h hUnc5B^Ecto^ was mixed with nanobody in 1:1, 1:5, and 1:10 M ratios in 20mM Tris-HCL, pH 7.5, 200mM NaCl, 1% BSA and incubated with hGPC3^core^-coated strep beads for 3 h. Beads were washed with 20mM Tris-HCL, pH7.5, 200mM NaCl and the proteins were eluted with SDS-containing gel-loading buffer supplemented with 5% beta-mercaptoethanol. Samples were analyzed using SDS-PAGE.

#### Cell morphology analysis

Nano^glue^ and Nano^break^ cloned into pCAGIG (Control) plasmids were electroporated at E13.5. After 3 days, embryonic brains were collected, fixed in 4% PFA overnight and vibratome cut into 75μm sections. Single cell morphology was reconstructed and analyzed using ImageJ (version 1.53) as described previously (Namba et al., 2014; del Toro et al., 2017). For single cell morphology analysis in the lateral cortex was used after maximum projection of a z stack representing 50-60um (one image per 5um). Single cell morphology from GFP-expressing neurons was reconstructed and analyzed using ImageJ (version 1.49). 15–20 neurons per cortical layer (upper CP, lower CP and IZ) were quantified per section (2-3 sections per brain and three independent brains per condition).

### Quantification and statistical analysis

Statistical analyses were performed using GraphPad Prism, employing a two-tailed unpaired Student’s *t* test (Figures 4J, 5D, 6D, S4J, and S7E) or chi-square contingency analysis (Figures 7D, 7F, and 7H) when comparing two groups or multiple groups distribution, and one-way ANOVA test with Tukey’s post hoc analysis when comparing multiple groups (Figures 3B, 3F, 3H, 4H, 4K, 6B, 6F, S3B, S3H, S4H, S6F, and S6H). p values represent ∗p ≤ 0.05, ∗∗p ≤ 0.01, ∗∗∗p ≤ 0.001 and ∗∗∗∗p ≤ 0.0001. All data are presented as the mean ± SEM, whisker plots or dot plots. All sample sizes and definitions are provided in the figure legends.

## Data Availability

• Crystallography data and models have been deposited at the PDB where they will be publicly available as of the date of publication. Accession numbers are listed in the key resources table. This paper also analyses existing, publicly available RNA sequencing data. Accession numbers are listed in the key resources table. • This paper does not report original code. • Any additional information required to reanalyze the data reported in this paper is available from the lead contact upon request.

## Supplemental Information

Document S1. Supplemental Alignment, related to Figure 1 Sequence alignment of Unc5 sequences, h: homo sapiens, m: mus musculus, g: gallus gallus, r: rattus norvegicus. Human Unc5A (isoforms1), which lacks the TSP1 domain, is indicated with an asterisks. Residues found in the GPC3 interacting interfaces 1–3 are highlighted by colored boxes. We used Clustal Omega (Madeira et al., 2022) and Espript (Gouet et al., 2003) to generate and visualise the alignment

Table S1. Crystallographic statistics, related to Figure 1
