# Supplementary material for: GPC3-Unc5 receptor complex structure and role in cell migration
Source: Cell. 2022 Oct 13;185(21):3931–3949.e26. doi: 10.1016/j.cell.2022.09.025 (PMC9596381; doi:10.1016/j.cell.2022.09.025)
Supplement: Document S1. Supplemental Alignment, related to Figure 1 — Sequence alignment of Unc5 sequences, h: homo sapiens, m: mus musculus, g: gallus gallus, r: rattus norvegicus. Human Unc5A (isoforms1), which lacks the TSP1 domain, is indicated with an asterisks. Residues found in the GPC3 interacting interfaces 1–3 are highlighted by colored boxes. We used Clustal Omega (Madeira et al., 2022) and Espript (Gouet et al., 2003) to generate and visualise the alignment [file mmc1.pdf]

Cell, Volume *185*

Supplemental information

GPC3-Unc5 receptor complex

structure and role in cell migration

Onno Akkermans, Céline Delloye-Bourgeois, Claudia Peregrina, Maria Carrasquero-Ordaz, Maria Kokolaki, Miguel Berbeira-Santana, Matthieu Chavent, Florie Reynaud, Ritu Raj, Jon Agirre, Metin Aksu, Eleanor S. White, Edward Lowe, Dounia Ben Amar, Sofia Zaballa, Jiandong Huo, Irene Pakos, Patrick T.N. McCubbin, Davide Comoletti, Raymond J. Owens, Carol V. Robinson, Valérie Castellani, Daniel del Toro, and Elena Seiradake

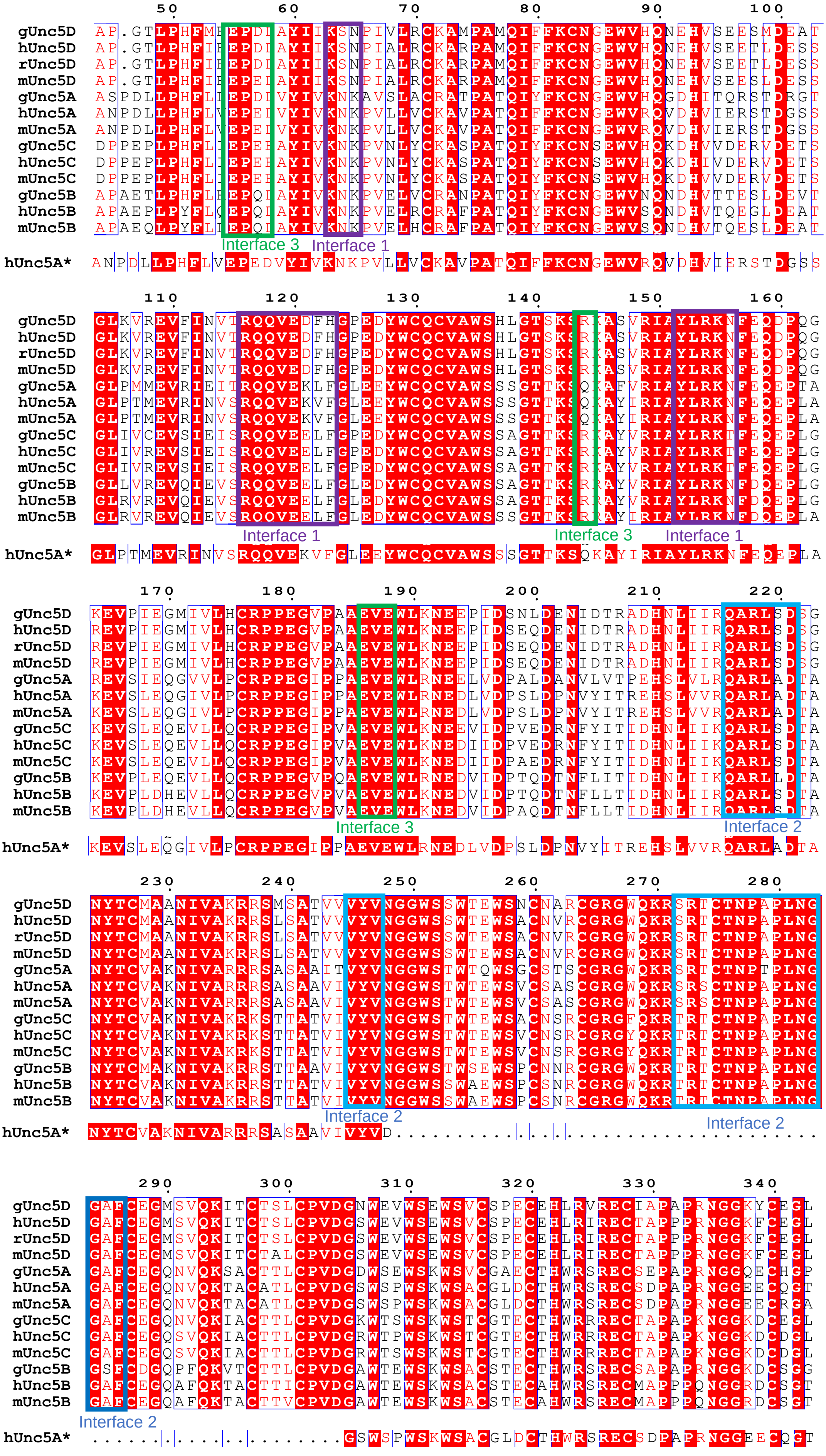
50 60 70 80 90 100
gUnc5D
hUnc5D
rUnc5D
mUnc5D
gUnc5A
hUnc5A
mUnc5A
gUnc5C
hUnc5C
mUnc5C
gUnc5B
hUnc5B
mUnc5B
Interface 3
Interface 1
hUnc5A*
110 120 130 140 150 160
Interface 1
Interface 3
Interface 1
170 180 190 200 210 220
Interface 3
Interface 2
230 240 250 260 270 280
Interface 2
Interface 2
290 300 310 320 330 340
Interface 2
