# Supplementary material for: GPC3-Unc5 receptor complex structure and role in cell migration
Source: Cell. 2022 Oct 13;185(21):3931–3949.e26. doi: 10.1016/j.cell.2022.09.025 (PMC9596381; doi:10.1016/j.cell.2022.09.025)
Supplement: Table S1. Crystallographic statistics, related to Figure 1 [file mmc2.pdf]

Table S1. Crystallographic statistics. Related to Figure 1.

**Data collection**					
Protein (PDB code)	hGPC3^{core} (7ZAW)	mGPC3^{core} (7ZAV)	hGPC3^{core} + rUnc5D^{Ig1Ig2TSP} (7ZA1)	mGPC3^{core} + rUnc5D^{IgIgTSP} (7ZA3)	mGPC3^{488} + rUnc5D^{IgIgTSP} (7ZA2)
Space group	P 3_1 2 1	P 3_1 2 1	P 1 2_1 1	P 3_1	P 1 2_1 1
Cell dimensions					
a, b, c (Å)<br>α, β, γ (°)	100.2, 100.2, 90.2<br>90, 90, 120	100.5 , 100.5, 90.6<br>90, 90, 120	102.6, 157.6, 126.6<br>90, 102.5, 90	119.6, 119.6, 257.9<br>90, 90, 120	102.1, 1580, 126.6<br>90, 102.9, 90
Resolution range, highest resolution shell (Å)	86.86-2.58 (2.65-2.58)	90.76-2.90 (2.98-2.90)	123.00-4.10<br>(4.55-4.10)	80.75-4.0<br>(4.23-4.0)	66.53-4.60<br>(4.76-4.60)
$R_{merge\ (\%)}$	7.5 (235.8)	13.0 (202.0)	10.9 (73.0)	5.3 (112.3)	9.28 (82.8)
I/σ(I)	16.0 (0.9)	9.2 (1.1)	3.4 (1.6)	12.6 (2.0)	7.77 (1.8)
Highest resolution shell with I/σ(I) > 2 (Å)	2.72-2.67	3.12-3.06	6.00-5.83	4.23-4.0	4.84-4.74
$CC_{1/2}$	99.9 (32.4)	99.7 (49.3)	99.4 (73.8)	100.0 (76.1)	99.7 (48.5)
Completeness (%)	100.0 (100.0)	100.0 (100.0)	99.9 (10.9)	73.8 (24.4)	99.4 (100.0)
Number of unique reflections	16895 (1239)	12106 (875)	18023 (900)	26038 (1287)	21870 (2165)
Redundancy	15.5 (6.9)	9.5 (9.6)	3.3 (3.5)	8.2 (7.7)	6.6 (6.4)
Wilson B (Å$^{2)}$	87.6	92.0	143.4	269.6	236.1
**Refinement**					
Resolution (Å)	50.10-2.58 (2.67-2.58)	43.98-2.90 (2.97-2.89)	97.20-4.10 (4.25-4.10)	80.90-4.00	66.60-4.60 (4.76-4.60)
R_{work}/R_{free*}	0.23/0.28	0.22/0.26	0.32/0.36	0.36/0.38	0.31/0.35
No of non-hydrogen atoms	2864	2786	19646	19445	19445
protein	2836	2758	19148	19188	19188
other	28	28	498	257	257
mean B-values (Å^2)	100.43	86.61	143.4	269.6	236
Ramachandran plot					
favoured (%)	95.99	96.76	96.10	96.69	96.69

outliers (%)	0.00	0.00	0.42	0.47	0.47
Bond length deviations (Å)	0.004	0.004	0.0079	0.0076	0.0079
Bond angle deviations (°)	0.690	0.670	1.572	1.375	1.384
